# Abiotic Factors and Plant Communities Shape the Distribution of Soil Pathogenic Oomycetes in Chinese Grasslands

**DOI:** 10.1002/advs.202501994

**Published:** 2025-06-04

**Authors:** Junsheng Ke, Chen Zhu, Peixi Jiang, Peng Zhang, Kui Hu, Yilin Dang, Yao Xiao, Mu Liu, Huiying Liu, Xiang Liu, Ville‐Petri Friman

**Affiliations:** ^1^ State Key Laboratory of Herbage Improvement and Grassland Agro‐Ecosystems & College of Ecology Lanzhou University Lanzhou Gansu 730000 China; ^2^ Key Laboratory of Biology Genetics and breeding of Special Economic Animals and Plants Ministry of Agriculture and Rural Affairs Tea Research Institute Chinese Academy of Agricultural Sciences Hangzhou 310008 China; ^3^ Xi hu National Agricultural Experimental Station for Soil Quality Hangzhou 310008 China; ^4^ Zhejiang Tiantong National Station for Forest Ecosystem Research The Shanghai Key Lab for Urban Ecological Processes and Eco‐Restoration School of Ecological and Environmental Sciences East China Normal University Shanghai 200241 China; ^5^ Institute of Eco‐Chongming (IEC) East China Normal University Shanghai 202162 China; ^6^ Department of Microbiology University of Helsinki Viikinkaari 9 Helsinki 00014 Finland

**Keywords:** climate change, disease risk, grassland ecosystem, soil oomycete pathogen

## Abstract

The outbreaks of emerging phytopathogens constrain socioeconomic development globally and are expected to intensify in the future along with climate change. Oomycetes, a group of fungus‐like eukaryotes, include many phytopathogenic species, making it critical to understand the drivers of their diversity and distribution. This work analyzes 972 soil samples from three major grassland types in China and found that soil phosphorus availability drove oomycete richness, while humidity and nitrogen content affected community composition. Pathogenic oomycete abundance is mostly influenced by precipitation, temperature seasonality, and plant species richness. This work creates a distribution atlas of pathogenic oomycete richness and abundance in Chinese grasslands, using space‐for‐time methods to predict future outbreak areas under climate change. Model predictions indicate a potential increased risk of oomycete disease in ≈42% of the grassland area under SSP 1–2.6 and SSP 5–8.5 climate scenarios, particularly in portions of typical and meadow grasslands. This study enhances the understanding of the drivers behind the distribution of pathogenic oomycetes and highlights the need for disease management strategies in the face of climate change.

## Graphical abstract

The continental‐scale study reveals that soil phosphorus strongly predicts the richness of soil pathogenic oomycetes in Chinese grasslands, while precipitation and plant species richness drive their absolute abundance; modeling predicts ≈42% of grassland areas may face elevated oomycete disease risk under future climate scenarios, especially in typical and meadow grasslands.

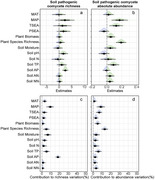

## 1 Introduction

The plant disease outbreaks caused by microbial pathogens disrupt food supply chains, threaten cotton and timber production, impact international trade, and jeopardize terrestrial ecosystem health and future food security.^[^ ^1^, ^2^ ^]^ The damage caused by plant pathogens has a huge impact on global crop production, resulting in annual yield reductions ranging from 10% to 20%.^[^ ^3^, ^4^ ^]^ Combined with previous studies, global climate change and intensified human activities are likely to further aggravate the emergence and spread of phytopathogens,^[^ ^5^, ^6^, ^7^, ^8^, ^9^ ^]^ resulting in increased crop losses in the future. While most research to date has focused on the impacts of pathogens in agricultural systems, plant pathogens are also a normal part of microbiota in natural ecosystems and play an essential role in inducing conspecific negative density dependence,^[^ ^10^, ^11^ ^]^ maintaining plant diversity,^[^ ^12^ ^]^ shaping biodiversity‐productivity relationships,^[^ ^13^ ^]^ and facilitating ecological succession.^[^ ^14^ ^]^ As natural ecosystems are more heterogeneous and diverse, they often also hold relatively higher diversity of phytopathogens,^[^ ^15^ ^]^ forming a potential reservoir from where they could spill over and colonize into agro‐ecosystems through natural dispersal, weather disruption or due to human activities.^[^ ^16^ ^]^ Understanding the distribution and drivers underlying plant pathogen richness, composition and abundance in natural ecosystems is hence critical for agroecosystem sustainability.^[^ ^7^, ^17^ ^]^

One important group of phytopathogens with both environmental and agricultural populations is oomycetes — a distinct group of fungal‐like eukaryotic microorganisms phylogenetically closely related to diatoms and brown algae.^[^ ^18^, ^19^ ^]^ Unlike fungi, oomycetes possess unique biological traits, such as cellulose‐based cell walls and diploid‐dominant life cycles, which not only distinguish them evolutionarily and functionally, but also shape their ecological interactions and responses to environmental gradients.^[^ ^20^ ^]^ These microorganisms also include several socioeconomically important pathogens such as *Phytophthora infestans* that caused the Irish Potato Famine.^[^ ^21^ ^]^ Several previous studies conducted at local spatial scales have mapped the distribution, diversity and abundance of fungal plant pathogens,^[^ ^10^, ^22^, ^23^ ^]^ in relation to both abiotic and biotic factors across various natural ecosystems and agroecosystems.^[^ ^8^, ^24^, ^25^, ^26^, ^27^ ^]^ However, a broader synthesis aiming to understand the patterns and potential underlying drivers of soil pathogenic oomycete distribution at broad geographic scales is still lacking, and understanding their unique ecological responses is essential for developing effective management and control strategies.

Based on the disease triangle concept, the interactions between pathogens, hosts and environment are crucial for the emergence of plant diseases,^[^ ^28^ ^]^ and several abiotic and biotic factors have been attributed to the structuring of soil pathogenic oomycete communities.^[^ ^29^, ^30^ ^]^ Water availability is one of the primary factors determining the distribution, diversity and abundance of soil oomycetes, since high humidity promotes the reproduction of most oomycetes by facilitating sporangial release, zoospore swimming and asexual sporangia production.^[^ ^20^, ^31^ ^]^ Moreover, oomycetes may be adapted to grow in a particular temperature range,^[^ ^32^ ^]^ as warmer environments are often beneficial for hyphae growth, while too high temperatures can be lethal due to drying of the soils they live in. Oomycete zoospores can encyst, allowing survival under unfavorable conditions until germination and production of hyphae once the surrounding environment regains adequate moisture and nutrient levels.^[^ ^20^, ^33^ ^]^ Notably, cell walls constitute a significant part of oomycete biomass, which is composed of cellulose and β‐glucans. As a result, soil properties, including carbon, nitrogen and phosphorus availability can significantly affect the growth and abundance of soil oomycetes.^[^ ^8^, ^34^ ^]^ Beyond these abiotic factors, co‐evolution with host plants can shape the composition and diversity of pathogenic oomycetes,^[^ ^35^ ^]^ similar to fungal pathogens.^[^ ^36^, ^37^ ^]^ For example, specialized pathogen strains can produce highly specific effector molecules that interact with host immune receptors or enzymes, resulting in host‐specific patterns of co‐evolution.^[^ ^38^ ^]^ The probability that oomycetes can infect two host plant species decreases rapidly along with their phylogenetic distance,^[^ ^39^ ^]^ and hence, plant species richness is likely to correlate with the richness of host‐specific oomycete pathogens.^[^ ^40^ ^]^ Moreover, some generalized pathogenic oomycetes can also increase their abundance by infecting a broader range of hosts and hence benefit from high plant diversity.^[^ ^41^, ^42^ ^]^ While previous research suggests that both abiotic and biotic factors drive the variation in the richness, composition and abundance of pathogenic oomycetes, most of this evidence comes from local case studies.^[^ ^30^, ^40^, ^42^ ^]^ We thus poorly understand how abiotic and biotic factors shape oomycete richness, composition and abundance across larger spatial scales. This information is especially important for predicting how global change and biodiversity loss will affect oomycete distribution and range expansion in the future.

Here, we conducted a large‐scale field survey of 972 soil samples from 244 natural grassland sites in China, covering three main grassland types (meadow grassland, typical grassland and desert grassland) spanning over 4000 kilometers. Grasslands cover 26% of the global terrestrial surface, play an essential role in biodiversity maintenance, climate regulation and carbon storage,^[^ ^43^ ^]^ and contain abundant pathogenic oomycetes that vary from biotrophs (e.g., *Albugo* and *Peronospora*) to hemibiotrophs (e.g., *Phytophthora*) and necrotrophs (e.g., *Pythium*). Field surveys across large spatial scales can hence provide an ideal system to disentangle the relative importance of abiotic and biotic factors in shaping the richness, composition and abundance of pathogenic oomycetes. To enable the identification of pathogenic oomycete taxa, we established an oomycete annotation database, *Oomydb*, including reference sequences and taxonomic information, as well as guild types at the genus level. By referencing to *Oomydb* database, a total of 1532 pathogenic oomycete zOTUs (Zero‐radius Operational Taxonomic Units) were identified using amplicon sequencing based on internal transcribed spacer 1 (ITS1) gene. Additionally, we constructed a plasmid with primers to quantify the absolute abundance of soil pathogenic oomycetes (see *Experimental Section* below) in each sample. By combining 18 biotic and abiotic factors obtained from databases and field surveys, we analyzed which factors best explained oomycetes richness, abundance and distribution, and then performed distribution projections with space‐for‐time methods to address the following questions: (i) Do richness and absolute abundance of pathogenic oomycetes differ between three grassland types (meadow grassland, typical grassland and desert grassland)? (ii) What underlying abiotic and biotic factors affect pathogenic oomycete richness, community composition and abundance? (iii) What will the predicted future distribution patterns of pathogenic oomycete richness and absolute abundance be based on future climate change scenarios (SSP 1–2.6 and SSP 5–8.5)?

## 2 Results

Based on the field survey across China's grasslands spanning 4000 kilometers, we first built a dataset of 2269 zOTUs to identify phytopathogenic oomycetes at the genus level with high confidence. These zOTUs belonged to 5 orders (mainly Peronosporales and Saprolegniales), 11 families (mainly Pythiaceae, Saprolegniaceae and Peronosporaceae) and 15 genera (mainly *Globisporangium*, *Saprolegnia*, *Phytophthora* and *Pythium*). Of all zOTUs, 1532 zOTUs (67.52%) were classified as “potential plant pathogenic” taxa based on oomycete annotation database (*Oomydb*), which we constructed (see *Experimental Section* below). These phytopathogenic taxa were mainly dominated by *Globisporangium* (48.4%), *Phytophthora* (20.3%) and *Pythium* (18.9%) (Table S2, Supporting Information). The richness (i.e., number of zOTUs) and absolute abundance (i.e., number of gene copies per gram of soil) of soil pathogenic oomycetes ranged from 2 to 135 zOTUs and 5 to 8 990 9538 gene copies/g soil, respectively, across all soil samples.

### 2.1 Impacts of Grassland Types on Soil Pathogenic Oomycetes

**Figure 1:**
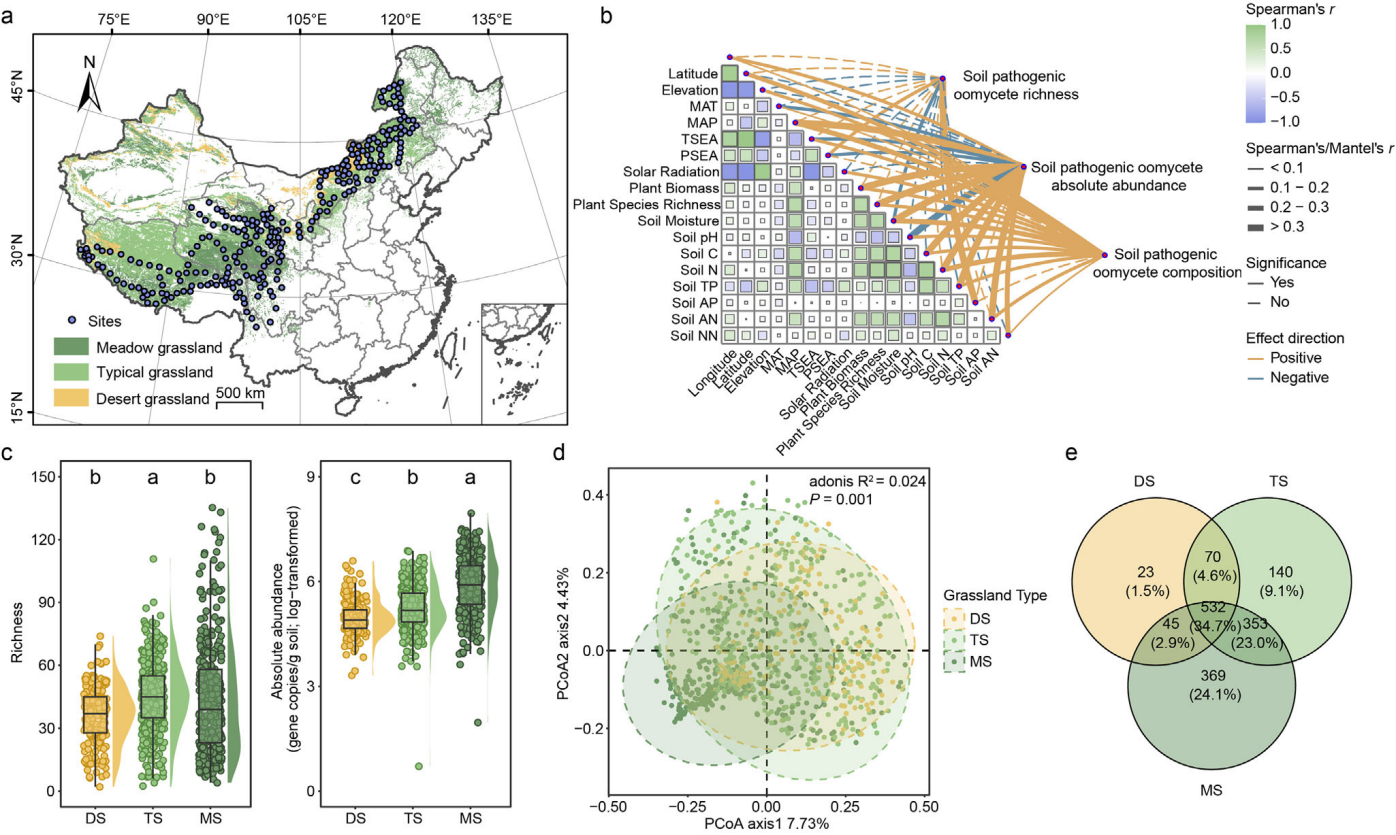
Geographic distribution of 244 study sites across three grassland types within China's main grasslands, and the relationships between 18 biotic and abiotic factors, including geomorphological and climatic factors, soil properties, plant community characteristics, and the richness, absolute abundance and community composition of pathogenic oomycetes. a) The figure presents the distribution of three natural grassland types (meadow grassland, typical grassland and desert grassland) in China based on the Albers projection. b) Size of the boxes in the figure represents the correlation coefficients between environmental variables, with green and blue indicating positive and negative relationships, respectively. The dotted lines in the figure represent non‐significant correlations between the richness/absolute abundance/composition of soil pathogenic oomycetes and the corresponding environmental factors. Green and purple solid lines indicate positive and negative correlations, respectively, between soil pathogenic oomycete richness/absolute abundance and environmental factors, with the width of lines reflecting the strength of Spearman's correlation analysis and Mantel test. The 18 biotic and abiotic variables include longitude, latitude, elevation, mean annual temperature (MAT), mean annual precipitation (MAP), temperature (TSEA) and precipitation seasonality (PSEA), solar radiation, plant species richness, plant biomass, soil moisture, soil pH, soil carbon (Soil C), soil nitrogen (Soil N), soil total phosphorus (Soil TP), soil available phosphorus (Soil AP), ammonia nitrogen (Soil AN) and nitrate nitrogen (Soil NN). c)The difference in pathogenic oomycete richness and absolute abundance between different grassland types (MS: meadow grassland, TS:typical grassland and DS:desert grassland) based on Mann‐Whitney U tests (Different letters indicate significant differences between the groups using letter notation). d) The principal coordinate analysis (PCoA) comparing differences in pathogenic oomycete community composition between three different grassland types (MS: meadow grassland, TS: typical grassland and DS: desert grassland). e) The Venn plot shows the counts of zOTUs that are shared among or unique to desert grassland (DS), typical grassland (TS) and meadow grassland (MS).

Given that varying attributes of different grassland types potentially influence soil pathogenic oomycetes, we compared their richness and absolute abundance between three grassland types. We found that richness (χ2 = 30.952, *p* < 0.001) and absolute abundance (χ2 = 226.08, *p* < 0.001) of soil pathogenic oomycetes were significantly different between grassland types: the highest pathogenic oomycete richness was observed in typical grasslands (the mean number of pathogenic oomycete zOTUs = 44.79 per sample), followed by meadow grasslands (the mean number of pathogenic oomycete zOTUs = 43.23 per sample) and desert grassland habitats (the mean number of pathogenic oomycete zOTUs = 36.05 per sample; **Figure** **1**c). In contrast to oomycete richness, absolute abundance of pathogenic oomycetes was the highest in meadow grasslands (the mean gene copies of pathogenic oomycete zOTUs = 3.13 × 10^6^ per gram soil), followed by typical grasslands (the mean gene copies of pathogenic oomycete zOTUs = 4.58 × 10^5^ per gram soil) and desert grassland habitats (the mean gene copies of pathogenic oomycete zOTUs = 2.31 × 10^5^ per gram soil). The community composition of pathogenic oomycetes also differed between different grassland types (*F*_2,969_ = 12.049, *p* < 0.001, *R* ^2^ = 0.024; Figure 1d). Meadow grasslands presented more unique pathogenic oomycetes than desert grasslands and typical grasslands (Figure 1e). Together, these findings suggest that Chinese grasslands harbor a high diversity of phytopathogenic oomycetes that vary depending on the grassland type.

### 2.2 Assessing the Relative Importance of Different Abiotic and Biotic Factors Associated with Soil Pathogenic Oomycetes in Grasslands

We next used the Spearman's rank correlations to assess the effects of geomorphologic and climate factors, soil properties and plant community characteristics on the richness and absolute abundance of soil pathogenic oomycetes across all grassland types. Overall, climatic factors, soil properties and plant community characteristics played important roles in shaping the richness and absolute abundance of soil pathogenic oomycetes (Figure 1b). Specifically, we found that soil available phosphorus was positively associated with soil pathogenic oomycete richness (Spearman's *r* = 0.38, *p* < 0.001; Figure 1b), especially in typical grasslands (Spearman's *r* = 0.29, *p* < 0.001) and meadow grasslands (Spearman's *r* = 0.62, *p* < 0.001), while mean annual precipitation, plant biomass, plant species richness and soil properties were positively associated with the absolute abundance of soil pathogenic oomycetes (Figure 1b). Furthermore, the Mantel test was used to explore the impact of multiple variables on the community composition of soil pathogenic oomycetes. The results showed that mean annual precipitation (Mantel's *r* = 0.21, *p* < 0.001), soil moisture (Mantel's *r* = 0.21, *p* < 0.001) and soil nitrogen (Mantel's *r* = 0.22, *p* < 0.001) clearly affected the community composition of soil pathogenic oomycetes across the whole data. While the typical and meadow grasslands exhibited relatively similar response patterns, the associations observed in desert grasslands were weaker, with only a few statistically significant relationships observed (Figures S4 and S7 and Tables S8 and S9, Supporting Information).

**Figure 2:**
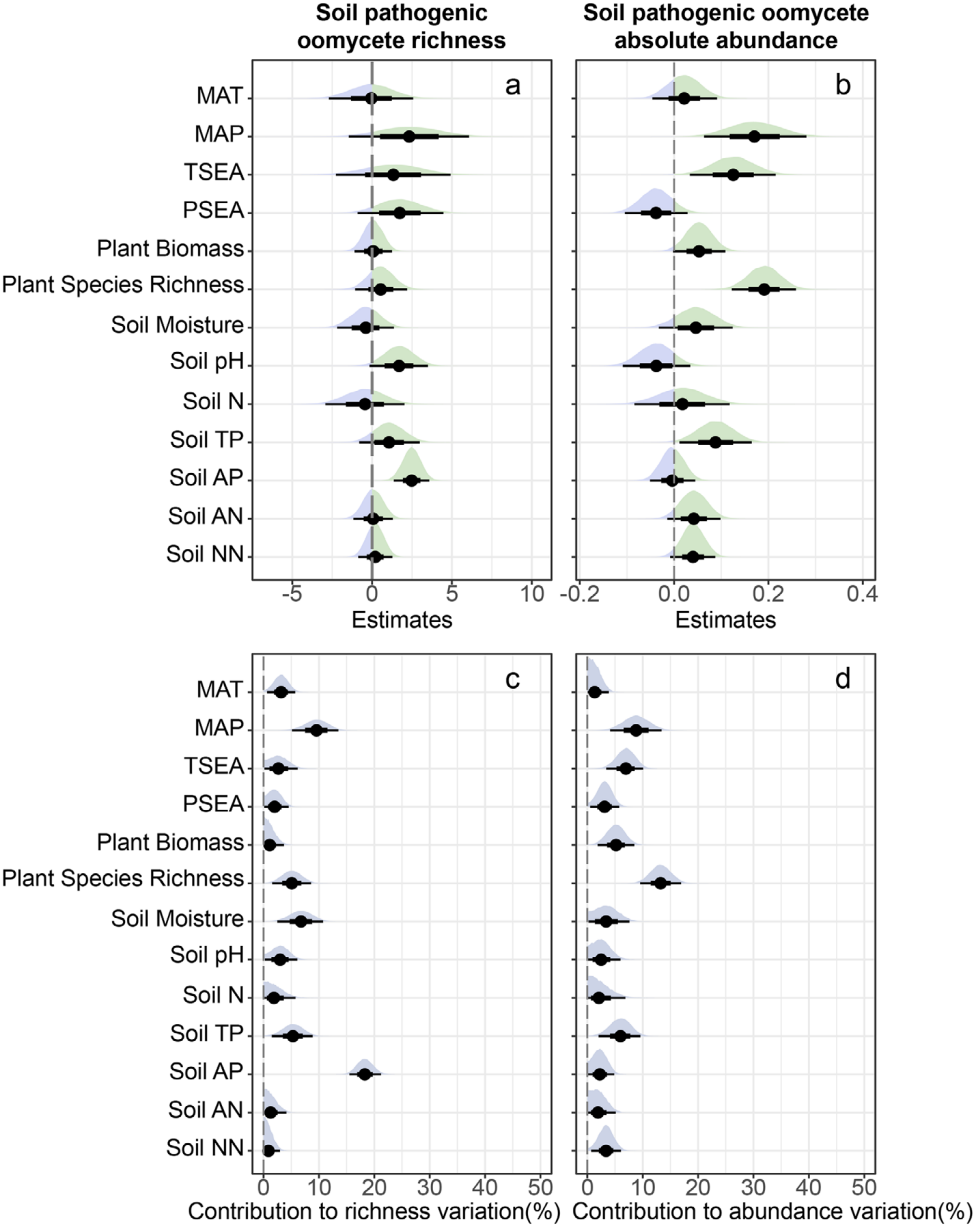
The effects and contribution of climatic factors, soil properties, plant community characteristics to richness and absolute abundance of pathogenic oomycetes. a) The effects on the richness of soil pathogenic oomycetes. b) The effects on the absolute abundance of soil pathogenic oomycetes. c) The contribution on the richness of soil pathogenic oomycetes. d) The contribution on the absolute abundance of soil pathogenic oomycetes. The distribution, mean, 50% and 95% confidence intervals for the values of each estimated effect and contribution are presented in the figure.

To distinguish the relative importance of different environmental variables in predicting the richness of pathogenic oomycetes across whole data, we employed a full Bayesian mixed‐effects model to examine how climatic factors, soil properties and plant community characteristics affected soil pathogenic oomycete richness. All environmental variables in Bayesian mixed‐effects models were tested for multicollinearity before inclusion in the model (Table S5, Supporting Information). Among these predictors, we found that only soil available phosphorus increased the richness of soil pathogenic oomycetes (slope = 2.48, 95% CIs = 1.35 to 3.58), explaining more than 18.3% of the total variation (**Figure** **2**a,c and Tables S5 and S6, Supporting Information). We further analyzed these effects for the dominant genera of soil pathogenic oomycetes and found that the richness of the three most abundant genera (i.e., *Globisporangium*, *Pythium* and *Phytophthora*) was positively associated with soil available phosphorus (Figure S6, Supporting Information). These results highlight the critical role of soil phosphorus availability in determining phytopathogenic oomycetes richness. However, other variables could not predict the variation of pathogenic oomycetes well, and the responses of major pathogenic oomycete genera were not consistent.

We next explored the effect of the same environmental variables on pathogenic oomycete absolute abundance. Based on Bayesian mixed‐effects model, we found that soil pathogenic oomycete absolute abundance was positively associated with mean annual precipitation (slope = 0.17, 95% CIs = 0.06 to 0.28), seasonality of temperature (slope = 0.13, 95% CIs = 0.03 to 0.22), plant species richness (slope = 0.19, 95% CIs = 0.12 to 0.26), plant biomass (slope = 0.05, 95% CIs = 0 to 0.11) and soil total phosphorus content (slope = 0.09, 95% CIs = 0.01 to 0.16) (Figure 2b and Table S5, Supporting Information). Among these variables, plant species richness explained most of the variation in phytopathogenic oomycete absolute abundance (13.24%), followed by mean annual precipitation (8.80%), temperature seasonality (6.91%), soil total phosphorus content (5.94%) and plant biomass (5.18%) (Figure 2d and Table S6, Supporting Information). Especially the abundance of most destructive oomycete genera, including *Phythium* and *Phytophthora*, showed strong and consistent correlations with plant species richness and mean annual precipitation (Figure S6, Supporting Information). To further assess the influence of climatic conditions during the year of sampling, we employed a linear mixed‐effects model and also observed a positive effect of precipitation during both sampling years (2021: *Z* = 2.850, *p* = 0.004; 2022: *Z* = 6.640, *p* < 0.001) on the absolute abundance (Figure S8 and Table S12, Supporting Information). Together, these results suggest that soil phosphorus was an important factor determining oomycete richness, while plant richness, precipitation and temperature seasonality were important in explaining highly pathogenic oomycete abundance across Chinese grasslands.

### 2.3 Direct and Indirect Influences of Abiotic and Biotic Factors on Soil Pathogenic Oomycetes

**Figure 3:**
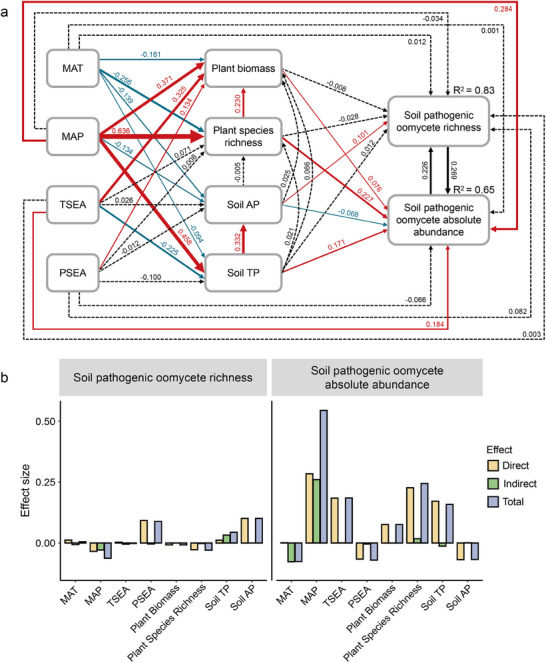
Results of the Bayesian structural equation models and the standardized effects of environmental variables on richness and absolute abundance of pathogenic **oomycetes**. a) The results reflect the influence of mean annual temperature (MAT), annual precipitation (MAP), temperature seasonality (TSEA), and precipitation seasonality (PSEA) in modulating the richness and absolute abundance of pathogenic oomycetes via plant biomass, plant species richness, soil available phosphorus (Soil AP), and soil total phosphorus (Soil TP). The coefficient estimates of the significant paths and the R‐squared values of the response variables are labeled in the figure. Green and purple arrows represent positive and negative effects, respectively, while grey dotted arrows represent non‐significant path coefficients. b) The effects (including direct, indirect, and total effects) of the variables involved in SEM on the richness; and absolute abundance of soil pathogenic oomycetes.

To reveal the potential direct and indirect effects of climatic, soil and plant factors on the richness and abundance of pathogenic oomycetes, we constructed Bayesian structural equation models based on our a priori knowledge (Figure S5, Supporting Information). The Bayesian structural equation model adequately fitted the data, with four split Markov Chain Monte‐Carlo chains at recommended convergence (Rhat = 1), and all posterior estimates showed well‐behaved distributions with acceptable uncertainty intervals (**Figure** **3**). By estimating the direct and indirect effects of each variable in standardized pathway coefficients of the Bayesian structural equation model, we detected soil available phosphorus (slope = 0.10, 95% CIs = 0.05 to 0.15) played an important role in explaining the variance in soil pathogenic oomycete richness (Figure 3a). Moreover, the mean annual precipitation increased soil pathogenic oomycete absolute abundance, through both direct (slope = 0.28, 95% CIs = 0.17 to 0.40) and indirect effects via plant biomass, plant species richness, soil available and total phosphorus (Figure 3). These findings suggest that climatic factors influenced pathogenic oomycete richness indirectly through soil available phosphorus, whereas their effects on pathogenic oomycete absolute abundance were mediated directly or indirectly, primarily through plant species richness.

### 2.4 Current and Future Distribution Projections of Soil Pathogenic Oomycetes in Chinese Grasslands

**Figure 4:**
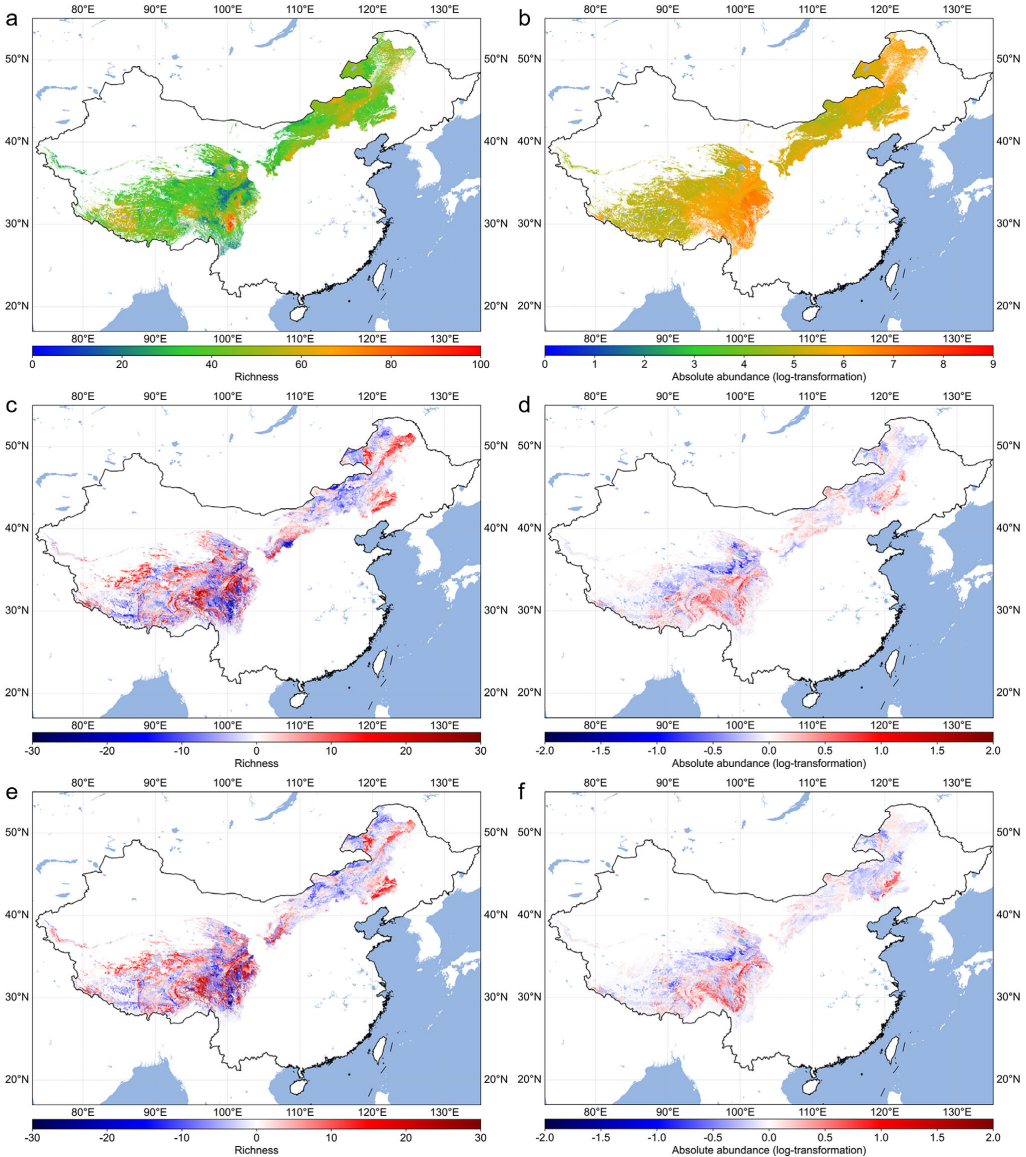
Current status and temporal projections (2040) of pathogenic oomycetes richness and absolute abundance (log‐transformed) across China's main grasslands. a) The projection of the current distribution for soil pathogenic oomycete richness. b) The projection of the current distribution for soil pathogenic oomycete absolute abundance. c) The changes in soil pathogenic oomycete richness between current and SSP 1–2.6 scenarios in 2040. d) The changes in soil pathogenic oomycete absolute abundance between current and SSP 1–2.6 scenarios in 2040. e) The change in soil pathogenic oomycete richness between current and SSP 5–8.5 scenarios in 2040. f) The change in soil pathogenic oomycete absolute abundance between current and SSP 5–8.5 scenarios in 2040. The prediction models were made using random‐forest analysis using the climate variables (mean annual temperature, annual precipitation, temperature and precipitation seasonality). The mapping areas include the main grassland regions in China, which are mainly distributed in the Qinghai‐Tibetan Plateau and Inner Mongolia.

As mean annual precipitation played a dominant role in predicting variation in the soil pathogenic oomycete absolute abundance (Figure 3), we used information on the current oomycete distribution in grasslands based on four climatic variables (MAT, MAP, TSEA, PSEA) to project their future richness and absolute abundance in China along with climate change. While soil available phosphorus was an important determinant of richness, climate variables were prioritized in projections due to their strong large‐scale influence and availability in future climate models. Currently, the area around western Sichuan harbors the highest level of soil pathogenic oomycete richness (**Figure** **4**a), whereas the eastern Qinghai‐Tibetan Plateau and the Greater Khingan Range have the relatively higher phytopathogenic oomycete absolute abundance (Figure 4b). Future projections were performed for soil pathogenic oomycete richness and absolute abundance in 2040 based on the climatic forecasts of the different shared socioeconomic pathway (SSP) scenarios 1–2.6 and 5–8.5 (represented sustainable and unsustainable future climate change scenarios, respectively) published by the Intergovernmental Panel on Climate Change (IPCC). Although the overall area of increasing oomycete richness expanded under both climate scenarios, an obvious increase was observed in meadow grasslands, particularly under the SSP 5–8.5 scenario (Figure S9, Supporting Information). ≈42% of the grassland areas were predicted to experience a potential increase in oomycete disease risk, with a notable portion of the increase occurring in typical and meadow grasslands. Surprisingly, the spatial extent of increased oomycete richness and absolute abundance in desert grasslands was greater under SSP 1–2.6 than under SSP 5–8.5. Moreover, we identified that Hulunbuir, the eastern Greater Khingan Range and Chifeng in Inner Mongolia, and eastern Qinghai‐Tibetan Plateau, could be facing significantly higher risks under global change as these areas are projected to have higher soil pathogenic oomycete richness and/or absolute abundance (Figure 4c–f).

## 3 Discussion

In this study, we identified 1532 zOTUs as potential plant pathogenic oomycetes, accounting for 67.52% of total zOTUs we found. The proportion of plant pathogenic oomycetes is much higher than that of fungi and bacteria,^[^ ^23^, ^44^ ^]^ which is closely related to their life history characteristics and their long‐term co‐evolution with host plants.^[^ ^28^ ^]^ Our results reveal that soil available phosphorus played a crucial role in driving soil pathogenic oomycete richness, which is different from fungal and bacterial pathogens whose abundance and distribution have been shown to be driven by temperature, precipitation, and soil pH.^[^ ^10^, ^25^, ^45^ ^]^ In contrast, the community composition of soil pathogenic oomycetes was regulated by water availability (i.e., mean annual precipitation and soil moisture) and soil nitrogen content, while absolute abundance was largely regulated by mean annual precipitation, temperature seasonality and plant species richness. This demonstrates that climatic factors, plant community characteristics and soil properties collectively drive the diversity, abundance and distribution of soil pathogenic oomycetes, reflecting the complex interactions between pathogenic oomycetes and biotic and abiotic factors in natural ecosystems.^[^ ^20^, ^46^ ^]^ Finally, we used the created database to predict oomycete's current distribution and response to two different climate change scenarios, which both suggested increased risk of disease outbreaks in the future. Together, our findings suggest that diverse and abundant phytopathogenic oomycete populations exist in natural grassland ecosystems which could form environmental reservoirs for pathogen spillover into agricultural environments.

Our results showed that soil pathogenic oomycete richness was positively associated with soil available phosphorus. Phosphorus is a vital component of oomycete cellular composition and also participates in the synthesis of different substances for metabolism (i.e., nucleic acid, phosphatase) and energy conversion (i.e., ATP, ATPases).^[^ ^47^ ^]^ Specifically, phosphorus is important for the synthesis of several virulence factors in oomycetes, including endocellulases, 1,3‐b‐glucanases, β‐glucosidases, cutinases, pectin‐esterases, galactanases, and endopolyga‐lacturonases.^[^ ^31^, ^48^ ^]^ Compared to soil carbon (especially organic carbon) and nitrogen, which generally reflect overall soil fertility or microbial biomass, available phosphorus is more responsive to better capture the immediate ecological conditions influencing pathogenic oomycetes.^[^ ^49^ ^]^ Its fast turnover and role in oomycete life activities may make it a more direct driver of oomycete diversity. The positive correlation is also in line with the results for pathogenic fungi,^[^ ^50^ ^]^ while a negative correlation between soil available phosphorus and the richness of mycorrhizal fungi has also been demonstrated;^[^ ^51^ ^]^ this opposing pattern may reflect differences in ecological processes and nutrient acquisition mechanisms between different functional groups. High phosphorus availability may also influence host plant quality and alter disease‐suppressive soil microbiome, thereby allowing a more diverse oomycete population to survive in natural populations.^[^ ^52^, ^53^, ^54^ ^]^ Surprisingly, plant species richness was a poor predictor of the variance in pathogenic oomycete richness, which is inconsistent with a previous multi‐vegetation‐pathogen study where the richness of soil pathogenic oomycetes was mainly explained by plant community characteristics.^[^ ^40^ ^]^ One likely explanation for this discrepancy could be the relatively broader plant host range of grassland soil pathogenic oomycetes compared to oomycetes found in monocultural agroecosystems, which tends to cause enrichment of specialized oomycete pathogens.^[^ ^27^ ^]^ The grassland ecosystems hold high plant species diversity (nearly 40 species within 1 square meter in some meadow grassland sites we sampled), where both specialist (i.e., *Peronospora*, *Albugo*) and generalist pathogenic oomycetes were commonly present.^[^ ^55^ ^]^ Specifically, we found that generalist oomycetes, including *Pythium*, *Phytophthora* and *Phytopythium*, dominated these communities. Therefore, it is not surprising that plant species richness was not a strong predictor of pathogenic oomycete richness in grassland ecosystems because as generalists they could likely infect multiple different plant species.^[^ ^36^ ^]^

Mean annual precipitation, plant species richness and soil properties could explain the community composition of pathogenic oomycetes. Overall, higher water availability could be indicative of the number of suitable environments available for oomycetes, hence improving their survival and colonization.^[^ ^56^ ^]^ While plant diversity did not explain oomycete diversity, we observed clear effects of plant species diversity on oomycete community composition. It is possible that the interactions between pathogenic oomycetes and plant communities may be more complex in wetter areas (e.g., western Sichuan), where intense plant‐soil feedbacks and negative density dependence driven by conspecific pathogens plays a critical role in also maintaining high plant diversity.^[^ ^57^, ^58^, ^59^, ^60^ ^]^ Moreover, the dominant taxa of pathogenic oomycetes identified in this study, including *Globisporangium*, *Pythium* and *Phytophthora*, responded positively to humidity, which further confirmed the hydrophilicity of oomycetes.^[^ ^46^, ^55^ ^]^ More work is required to try to disentangle the direct and indirect roles of soil moisture on phytopathogenic oomycetes and their plant host distribution in the future.

We found that mean annual precipitation, temperature seasonality, soil total phosphorus, plant biomass and plant species richness were all positively associated with the absolute abundance of soil pathogenic oomycetes. These patterns are in line with previous studies, which have suggested that climatic variables are essential drivers of fungal disease prevalence at both local and global scales.^[^ ^25^, ^61^ ^]^ The absolute abundance of phytopathogens are generally considered to be more closely related to the disease prevalence, while several global analyses have also identified climate‐driven patterns explaining pathogen abundance variation.^[^ ^10^, ^24^ ^]^ Mechanistically, an increase in air humidity or soil moisture promotes the growth of hyphae and proliferation of oomycete spores and such growth‐promoting effects are especially noticeable for hydrophilic oomycetes.^[^ ^7^, ^47^, ^62^ ^]^ Similar to a global study demonstrating that temperature is a dominant driver in predicting relative abundance of soil pathogenic fungi,^[^ ^10^ ^]^ we found that seasonal fluctuations in temperature (i.e., temperature seasonality) positively associated with absolute abundance of soil pathogenic oomycetes. This may be attributed to the fact that relatively broader temperature range during the plant growth seasons might increase the colonization opportunities for oomycetes and reduce the risks of local extinctions^[^ ^55^ ^]^ – an example of potential storage effect.^[^ ^63^, ^64^ ^]^ This could be further promoted by the oomycetes’ ability to form cysts or chlamydospores that allow them to survive in a dormant state during unsuitable growth conditions.^[^ ^20^, ^46^ ^]^ Overall, oomycete abundance was predicted by multiple biotic and abiotic factors that likely had direct effects on the oomycetes and their associated host plants.

Plant‐pathogen interactions are located at the center of disease ecology,^[^ ^28^ ^]^ and plant pathogens largely depend on plant diversity and biomass for their growth and survival.^[^ ^22^ ^]^ In line with this, we found that the absolute abundance of pathogenic oomycetes was positively associated with plant biomass and plant species richness. According to the trophic cascade hypothesis,^[^ ^65^ ^]^ higher plant biomass also supports more pathogens by providing more resources to lower trophic levels.^[^ ^66^ ^]^ The positive association between plant species richness and absolute abundance of soil pathogenic oomycetes may also be due to provision of a higher number of specialized ecological niches for pathogenic oomycetes.^[^ ^30^, ^40^, ^67^ ^]^ Higher plant diversity can support a wider range of pathogenic taxa, thus facilitating the proliferation of pathogenic oomycetes at the community level. Increased plant diversity may also enhance overall root biomass and rhizosphere complexity, creating more microhabitats and resource niches favorable for oomycete survival and growth.^[^ ^68^ ^]^ Furthermore, climate factors can also indirectly regulate pathogenic oomycetes by altering plant communities.^[^ ^69^ ^]^ By constructing Bayesian mixed‐effects models and structural equation models, we found that the associations of climatic factors and plant community characteristics on soil pathogenic oomycete richness were surprisingly insignificant compared to soil available phosphorus, whereas mean annual precipitation could largely explain the variations in absolute abundance through both direct and indirect pathways. Specifically, precipitation was positively associated with the plant species richness (consistent with a previous study across China's grasslands),^[^ ^70^ ^]^ which indirectly increased the absolute abundance of pathogenic oomycetes. Hence, in addition to the direct effects of climatic factors, the indirect climate effects on pathogenic oomycetes via plant communities should also be taken into consideration when assessing disease risks.^[^ ^40^ ^]^ While experimental work is required to causally demonstrate such links, our study suggests that SEM could be an useful way to identify potential causal relationships and associations between different variables.

Moreover, our results indicate that grassland types exhibit distinct oomycete distribution patterns and responses to environmental factors. In particular, the weaker and less consistent responses were observed in desert grasslands, which may be attributed to different plant characteristics and resource constraints.^[^ ^71^ ^]^ For example, low plant diversity and biomass typical for desert grasslands likely constrains oomycete host availability, thereby weakening plant‐pathogen interactions.^[^ ^59^ ^]^ Moreover, drought and resource scarcity may directly limit oomycete survival and activity, resulting in diminished ecological sensitivity.^[^ ^72^ ^]^ These results highlight the importance of considering habitat‐specific conditions when assessing the ecological risks posed by soil‐borne pathogens. Our analyses included a limited set of environmental variables, which may introduce some uncertainty due to lack of information on microclimatic and extreme weather effects.^[^ ^7^ ^]^ Incorporating these environmental and their potential interactions in the future studies may help better capture regional ecological oomycete density and community dynamics.

Most previous studies have focused on the relative abundance of plant pathogens,^[^ ^10^, ^23^ ^]^ even though pathogen absolute abundance could be considered a more meaningful measure from an ecological perspective, allowing quantification of actual exposure and disease risks in ecosystems.^[^ ^25^, ^73^ ^]^ While various (generalized) linear models have been employed to analyze distribution patterns along environmental gradients, these models often only reflect the overall driving effect of the environment and lack the synergistic effects between different variables.^[^ ^30^ ^]^ More advanced analytical methods, such as machine learning, have emerged to help us predict oomycete distribution patterns more intuitively and comprehensively in the face of climate change from different spatial and temporal perspectives,^[^ ^10^, ^23^, ^74^ ^]^ which provided practical guidance for preventing the oomycete disease outbreak in grasslands. Currently, locations with high precipitation tend to have higher risks of pathogenic oomycete outbreaks, and these risks are likely to become more intense in the future, which could pose risks for food production as these areas are highly productive and good‐quality pasture lands.^[^ ^75^ ^]^ While climate change could have strong local effects, for example, via increased precipitation that favor oomycete survival and reproduction, it could also shift the local distribution of pathogenic oomycetes, enabling them to expand their range to previously unsuitable areas.^[^ ^76^ ^]^ For example, although SSP 1–2.6 represents a sustainable development scenario, it projects greater increases in precipitation in arid grasslands compared to SSP 5–8.5, which could promote the expansion of pathogenic oomycetes in those regions. However, under the SSP 5–8.5 scenario, more meadow and typical grassland areas are projected to experience even higher increases in precipitation than under SSP 1–2.6, thereby further elevating the risks of oomycete‐related diseases. Climate change‐associated factors could hence promote pathogen spillover from natural to agricultural ecosystems, resulting in catastrophic disease outbreaks and reducing the productivity of agricultural and animal husbandry related activities.^[^ ^76^ ^]^

Together, our large‐scale survey across China's main grasslands highlights the critical and delicate effects of abiotic factors and plant communities in shaping the diversity, abundance and distribution of phytopathogenic oomycetes. Specifically, by creating an atlas of current distribution, abundance and diversity of oomycetes in China, we were able to predict the potential future risks of oomycete‐ associated plant diseases under projected climate scenarios, with higher levels in most fertile grasslands (typical and meadow grasslands) in China. These results highlight the urgency for adapting grassland agricultural management and pathogen control strategies for the future to maintain food security in the face of global climate change.^[^ ^77^ ^]^

## 4 Experimental Section

### Sampling Design and Data Collection

A large‐scale survey across China's main grasslands, covering most parts of Qinghai‐Tibet Plateau and Inner Mongolia Plateau was conducted from mid‐July to mid‐August between 2021 and 2022. Given the broad geographic scope and large number of sampling sites, sampling spanned two consecutive years. To minimize potential biases associated with phenological differences or climatic variation across years, the survey was carefully scheduled and conducted simultaneously along multiple parallel routes, ensuring that sampling in each region was completed within a relatively narrow time window at the peak of the growing seasons.^[^ ^78^ ^]^ A total of 972 samples originating from 244 study sites were collected, which covered a latitudinal gradient of ≈26.98°–50.15°N, a longitudinal gradient of ≈79.03°‐121.50°E, and spanned an elevational gradient of ≈403–4961 m a.s.l. (Figure 1a). Mean annual temperature and precipitation at the study sites ranged between ≈−5.7–10.0 °C and ≈27–924 mm, respectively. These geographical and climatic gradients were chosen based on previous studies reporting significant variation in pathogenic oomycetes richness and absolute abundance.^[^ ^79^, ^80^ ^]^ The exact study sites were selected based on no visible signs of cultivation, intensive grazing, clipping or other human activities, and based on their location far away from any urban areas. Chosen sites were located at least 500 meters from the closest main road and a minimum 50 kilometers from each other to ensure even distribution and independence of each study site.

For each site, we established four replicate sampling quadrats of 0.5 × 0.5 m (0.25 m^2^) at the corner of a 50 × 50 m sampling plot. Geographical information (i.e., longitude, latitude, elevation) for each quadrat was collected by using a GPS element (DCM84B, Dongmei Measuring Instruments Ltd., China). In each quadrat, we recorded the number of different plant species (plant richness) and clipped and collected plant biomass at the species level. Five soil cores (5 cm diameter and 15 cm depth) were collected arbitrarily and combined from each quadrat, targeting the soil layer with high microbial activity, including oomycetes, and frequent plant–microbe interactions.^[^ ^31^, ^40^ ^]^ Soil cores were subsequently placed in an icebox for refrigeration and a total of 972 soil samples were collected. Upon arrival at the laboratory, soil samples were immediately sieved (2 mm mesh) and split into three subsamples. One subsample was frozen at ≈20 °C for metabarcoding sequencing, the second subsample was used for analysis of soil physicochemical properties, and the third subsample was set aside at ≈80 °C for storage in case of verification.

### Quantification of Environmental Variables at Sampling Sites

A total of 18 biotic and abiotic variables were measured and used for the following analyses based on their potential for predicting pathogenic oomycete richness and absolute abundance (Table S1, Supporting Information).^[^ ^27^, ^30^, ^40^ ^]^ Geomorphologic and climatic factors were recorded at the site level, while soil properties and plant community characteristics were measured for each quadrat.

To collect climatic data at each site, we obtained mean annual temperature (MAT), annual precipitation (MAP), and temperature and precipitation seasonality (TSEA and PSEA) data from the WorldClim database v2.1 (http://www.worldclim.org; ≈1 km resolution) based on the coordinates of each site;^[^ ^81^ ^]^ this data reflects the long‐term seasonal changes in climatic conditions for each study site. Given the potential interannual climate variability, we extracted temperature and precipitation data for the sampling year (2021–2022) from the FLDAS Noah Land Surface Model L4 to support the interpretation of the results.^[^ ^82^ ^]^ Additionally, we included net downward shortwave radiation data from FLDAS database to account for the potential influence of elevated solar radiation on microorganisms in certain sampling regions, such as the Tibetan Plateau.^[^ ^83^ ^]^

Plant community characteristics included plant species richness and aboveground biomass. The aboveground plant biomass was determined at species level for all quadrats and plant species identified using the “Flora of China” identification guide.^[^ ^84^ ^]^ The number of different plant species detected at each quadrat was used as plant species richness index. After drying the aboveground plant samples in incubators at 65 °C for 72 h, the weight of each species for all quadrats was weighed to the nearest 0.1 mg. For each quadrat, the total dry weight of all plant species was calculated as the plant biomass.

All soil samples were analyzed using standardized protocols to avoid any bias between research laboratories, followed the methods described in Hu et al.^[^ ^85^ ^]^ To characterize the soil fertility at each location, we measured eight soil properties: soil moisture (%), soil pH, soil carbon (%; Soil C), soil nitrogen (%; Soil N), soil total phosphorus (‰; Soil TP), soil available phosphorus (mg kg^−1^; Soil AP), soil ammonia nitrogen (mg kg^−1^; Soil AN) and soil nitrate nitrogen (mg kg^−1^; Soil NN). The rest of the sieved soil was divided into two parts; one part was air‐dried and stored for a month to analyzes for soil moisture, soil pH, soil carbon, soil nitrogen, soil total and available phosphorus, the other was stored at −20 °C for determination of nitrogen in different forms. Pristine sieved soil and dried soil was weighed for moisture measurement; soil pH was determined with a pH meter in a 1:5 mass‐to‐volume soil‐water suspension; soil carbon and nitrogen content were determined using an elemental analyzer (Elementar vario MACRO cube, Elementar, Germany); soil total phosphorus was analyzed by molybdenum‐antimony colorimetry with acid digestion, soil available phosphorus by sodium bicarbonate leaching, and ammoniacal nitrogen and nitrate nitrogen by potassium sulphate leaching, which were all finally measured on a SmartChem 450 Automatic Discrete Chemistry Analyzer (SmartChem Technologies Ltd, Italy).

### Oomycetes Abundance Quantification with Plasmid and Sequencing

This work constructed a plasmid with primers targeting internal transcribed spacer 1 (ITS1) to quantify soil oomycetes abundance. ≈160 bp artificial DNA fragment was synthesized and ligated with forward and reverse oomycete‐specific primers (Forward primer OOMUP18Sc: 5′‐ TGCGGAAGGATCATTACCACAC ‐3′, and reverse primer ITS2‐OOM: 5′‐ GCAGCGTTCTTCATCGATGT ‐3′) at both fragment ends. OOMUP18Sc and ITS2‐OOM were used to amplify the ITS1 rRNA region of oomycetes.^[^ ^86^, ^87^, ^88^ ^]^ The fragment with ligated primers was then inserted into the EcoRV restriction enzyme site of the vector pUC57‐Amp (GENEWIZ, China) after 50 ul of plasmid solution with known concentrations (≈1.25 pg uL^−1^) was mixed with 500 mg of the soil to create a quantitative standard, the concentration was measured by a NanoDrop Microvolume Spectrophotometer (NanoDrop One, Thermo Fisher Scientific Inc., USA). Considering that the loss of plasmid and soil microbial DNA was equivalent during the extraction and amplification, we adjusted the number of sequenced oomycete reads with the number of artificial plasmid fragment reads during bioinformatics analysis (see *Bioinformatics analyses* below) to quantify absolute oomycete abundance in soil samples.

Total genomic DNA and plasmid from each sample was extracted using FastDNA SPIN Kits for Soil (MP Biomedicals, USA) according to the manufacturer's protocol. The fragments were amplified using polymerase chain reaction (PCR) in two steps, the first PCR step for amplifying the ITS1 target fragments and the second was Solexa PCR for ligating the bridging sequences, which were required to identify the samples in sequencing. The first PCR reaction for the ITS1 fragment was performed with the following mixture per sample: 50 ng of DNA template, 0.3 ul of each ITS primer (10 uM), 5 ul KOD FX Neo Buffer, 2 ul of dNTP (2 mM each), 0.2 ul of KOD FX Neo enzyme (TOYOBO, Japan), and ddH2O to reach a final volume of 10 ul. The PCR programme followed: 1 cycle of 95 °C for 5 min, followed by 25 cycles of 95 °C for 30 s, 50 °C for 30 s and 72 °C for 40 s, and a final extension step of 72 °C for 7 min. First PCR products were cleaned using VAHTS DNA Clean Beads (Vazyme, China). To ligate the bridges sequences, the solexa PCR was performed using the following 20 ul mixture per sample: 5 ul of first PCR cleaned products, 2.5 ul of MPPI‐a (2 uM), 2.5 ul of MPPI‐b (2 uM) and 10 ul of 2* Q5 High‐Fidelity 2* Master Mix (NEB, England). The solexa PCR programme was as follows: 1 cycle of 98 °C for 30 s, followed by 10 cycles of 98 °C for 10 s, 65 °C for 30 s and 72 °C for 30 s, and a final extension step of 72 °C for 5 min. Final PCR products were visually evaluated using gel electrophoresis (1.8% agarose gels) to ensure successful DNA amplification. DNA libraries for each sample were created and then paired‐end sequenced using Illumina Novaseq 6000 at Biomarker Technologies Co., Ltd. (Beijing, China). The general process of microbial quantification and sequencing is shown in Figure S1, Supporting Information, and the raw reads for all samples were deposited in the China National Center for Bioinformation database (CNCB) under the accession numbers PRJCA038309.

### Establishment of Oomycetes Annotation Database – *Oomydb*

This work established a reference sequence database for molecular identification of oomycetes called *Oomydb*, which was based on the nuclear ribosomal internal transcribed spacer (ITS) region and included ≈26 000 oomycete sequences with taxonomic and publication information. Oomycetes ITS sequence data were sourced from the Nucleotide Sequence Database of the National Center for Biotechnology Information (NCBI, accessed 25.02.2023) to cover all possible ITS sequences of oomycetes using the following search terms: oomycetes[ORGN] AND (internal transcribed spacer[TITL] NOT uncultured[TITL]).^[^ ^89^ ^]^ Raw sequences were identified and dereplicated, and one unique sequence with the longest fragment length was preserved. After removing the non‐oomycete sequences (e.g., Ascomycota, Basidiomycota), we corrected the taxonomic lineages based on authoritative classification systems. The NCBI Taxonomy classification was mainly used as the taxonomic backbone for *Oomydb* by default, and supplemented with updated information from the Global Biodiversity Information Facility (GBIF; https://www.gbif.org/) and Index Fungorum (http://www.indexfungorum.org/).^[^ ^90^ ^]^ *Oomydb* was applied to the annotation and bioinformatics analyses of the discovered oomycete sequences in this study.

### Bioinformatic Analyses

This work assembled paired‐end reads under the maximum number of 12 bp mismatches for long overlaps using USEARCH version 11.0.667,^[^ ^91^ ^]^ and trimmed off both forward and reverse primer complements before matching merged sequences against *Oomydb* database.^[^ ^92^ ^]^ The sequences with more than 1.0 expected error and < 160 bp were removed, and remaining sequences were truncated to 160 bp in length. Singletons and chimeric sequences were eliminated, and all sequences that passed the quality criteria were kept and assembled into zOTUs with 100% identity using UNOISE3 with default parameters.^[^ ^93^ ^]^ The taxonomic classification of each zOTU was determined by comparing against the *Oomydb* annotation database using the SINTAX function with a bootstrap cutoff of 0.6.^[^ ^94^ ^]^

This work obtained a total of 7 456 2072 high‐quality ITS rRNA gene reads and 31 189 zOTUs across 972 samples, with the abundance of reads per sample ranging from 31 084 to 117 505. Almost a third of all of zOTUs (29.6%) could be assigned at the family level, 7.3% at the genus level and only 4.6% were classified at the species level. The composition of oomycete communities based on the order level is shown in the Figure S3, Supporting Information. Oomycetes from the orders Albuginales and Peronosporales were classified as important plant pathogens, and detailed information of all potential plant pathogenic oomycetes with names and references at the genus level are listed in Table S2, Supporting Information. The number of observed plant pathogenic zOTUs was used as a measure of species richness and the absolute abundance of potentially plant pathogenic oomycetes was calculated by dividing the number of their reads by the number of plasmid reads in the same sample, and multiplying by the actual reads of plasmid added into soil samples. We generated rarefaction curves for all samples to assess and normalize sequencing depth (Figure S2, Supporting Information), using the function “*rarecurve*” in the R package “*vegan*.” ^[^ ^95^ ^]^ As the total absolute abundance of potential plant pathogenic oomycetes calculated by the un‐rarefied zOTU table was highly correlated with the same variable calculated by the rarefied zOTU table (rarefying to a minimum reads number of 31 084; Pearson's *r* = 0.999, *p* < 0.001), thus the un‐rarefied zOTU table was chosen to calculate richness and absolute abundance, in an attempt to generalize the variation in the overall pathogenic oomycete community. Meanwhile, the absolute abundance of soil pathogenic oomycetes was log‐transformed to improve the normality of residuals in statistical analyses.

### Statistical Analyses

To compare the richness and absolute abundance of soil pathogenic oomycetes in different grassland types, this work conducted the Kruskal‐Wallis test and Mann‐Whitney U tests to examine the differences among grassland types. This work then used a permutational multivariate analysis of variance (PERMANOVA) to test whether the composition of pathogenic oomycetes differed across grassland types. Further, Spearman's rank correlation analysis was used to analyze the relationship between plant pathogenic oomycete richness and abundance in relation to geomorphologic and climate factors, soil properties and plant community characteristics. The strength and direction of associations between two ranked variables were measured using the “*r.corr*” function in the R package “*Hmisc*.” ^[^ ^96^ ^]^ This analysis was chosen as it does not require the data to be linearly or normally distributed. This work also assessed the association between environmental variables and the richness and absolute abundance of the top ten plant pathogenic oomycetes genera (i.e., *Globisporangium*, *Pythium*, *Phytophthora*, *Pseudoperonospora*, *Hyaloperonospora*, *Bremia*, *Peronospora*, *Phytopythium*, *Elongisporangium*, *Pythiogeton*; Figure S5, Supporting Information).

To examine the relationships between biotic and abiotic factors and community composition of plant pathogenic oomycetes, this work generated a zOTU table for oomycetes across all samples and used Mantel test to calculate the matrix correlations between explanatory variables and community matrix using the “*ggcor*” function.^[^ ^97^ ^]^ The visualization of the results was integrated with the Spearman's rank correlation analysis.

This work further established Bayesian mixed‐effects models to compare the relative importance of abiotic and biotic factors in driving plant pathogenic oomycete richness and absolute abundance, using the R package “*brms*.” ^[^ ^98^ ^]^ All variables were standardized (Mean = 0, SD = 1) to allow direct comparison of regression coefficients. Before Bayesian mixed‐effects modeling, this work checked the multicollinearity between all included variables based on variance inflation factors (VIF < 10), and geographic variables, solar radiation and soil carbon content with highly collinearity were removed. In addition, this work constructed a spatial distance correlation matrix based on pairwise geographic distances derived from site coordinates using a Gaussian decay method,^[^ ^99^ ^]^ and included it with “*Site*” as a random effect in the models. In each model, the posterior distribution was sampled with four independent Markov Chain Monte‐Carlo (MCMC) chains with 10 000 iterations each, with the first 5000 skipped as burn‐in. The adapt‐delta value, average acceptance probability of target proposals was set as 0.99 to avoid divergent transitions after warm‐up. The convergence of the Markov chains was tracked by trace plots of posterior samples and when the Gelman‐Rubin convergence statistic (R‐hat) was < 1.01, the parameter estimates were considered to have converged to obtain estimated coefficients and 95% CIs of fixed effects in full models.^[^ ^100^ ^]^ This work also calculated variation in plant pathogenic oomycete richness and absolute abundance explained by environmental variables and error, to clarify the relative importance of variables in predicting potentially pathogenic oomycetes.

Variables that had significant effects in Bayesian mixed‐effects modeling (i.e., MAP, TSEA, plant biomass, plant species richness, soil TP, soil AP) were chosen to construct structural equation models. Temperature and precipitation were considered as fundamental indicators that shape the local plant and microbial communities and can provide basic information for local climatic conditions,^[^ ^24^, ^57^ ^]^ thus we took all four climatic indicators (MAT, MAP, TSEA, PSEA) into consideration. This work assumed a hypothesized prior model with these variables and constructed a structural equation model based on a Bayesian method to explore and evaluate the direct and indirect effects (via plant community characteristics and soil properties) of climate factors on soil pathogenic oomycetes. Standardized path coefficients and 95% CIs in the model were obtained to characterize the effect size and direction of each path. R‐squared values (var_fixed effects_/ var_fixed effects_ +var_residual effects_) were calculated using the “*bayes_R2*” function.^[^ ^101^ ^]^ All models were fitted using Stan and its interface with R in the “*brms*” package.^[^ ^98^ ^]^

### Mapping Current and Predicting Future Oomycetes Distribution

This work employed space‐for‐time methods to quantitatively predict the current and future distribution, richness and absolute abundance of plant pathogenic oomycetes in grasslands of China. Predictions on oomycete community composition were not included in the projections, as high spatial turnover and taxonomic uncertainty among oomycete species present challenges for reliable prediction at broad geographic scales.^[^ ^30^, ^42^ ^]^ Given that climate is the dominant driver shaping vegetation and environment, and that predictions of climatic data at fine spatial scales can be easily accessed under climate change scenarios, this work focused on changes in the four climatic variables (i.e., MAT, MAP, TSEA, PSEA) in predicting changes in oomycete richness and absolute abundance. This work extracted raster data of China's main grassland areas (mainly the Tibetan Plateau and Inner Mongolia), and estimated the current and future distribution of soil pathogenic oomycetes in the region under the two climate change scenarios. This work obtained multiple‐year (2021–2040) climate projection data under two different climate change scenarios (i.e., SSP 1–2.6 and SSP 5–8.5, assuming sustainable and unsustainable development scenarios, respectively) from the WorldClim database at a spatial resolution of 30 arc‐seconds73 [https://www.worldclim.org/], including four climate indicators mentioned above. All maps were visualized in Python 3.11.

## Conflict of Interest

The authors declare no conflict of interest.

## Supporting information



Supporting Information

## Data Availability

The data that support the findings of this study are openly available in [figshare] at [https://doi.org/10.6084/m9.figshare.27930840.v4], reference number [27930840].
