# Supplementary material for: Abiotic Factors and Plant Communities Shape the Distribution of Soil Pathogenic Oomycetes in Chinese Grasslands
Source: Adv Sci (Weinh). 2025 Jun 4;12(32):e01994. doi: 10.1002/advs.202501994 (PMC12407259; doi:10.1002/advs.202501994)
Supplement: Supplementary file 1 — Supporting Information [file ADVS-12-e01994-s001.docx]

Supporting Information

**Abiotic Factors and Plant Communities Shape the Distribution of Soil Pathogenic Oomycetes in Chinese Grasslands**

*Junsheng Ke, Chen Zhu, Peixi Jiang, Peng Zhang, Kui Hu, Yilin Dang, Yao Xiao, Mu Liu, Huiying Liu, Xiang Liu*, Ville-Petri Friman*

**Affiliations:**

^1^*State Key Laboratory of Herbage Improvement and Grassland Agro-Ecosystems & College of Ecology, Lanzhou University, Lanzhou, Gansu 730000, China*

^2^*Key Laboratory of Biology, Genetics and breeding of Special Economic Animals and Plants, Ministry of Agriculture and Rural Affairs, Tea Research Institute, Chinese Academy of Agricultural Sciences, Hangzhou 310008, China*

^3^*Xihu National Agricultural Experimental Station for Soil Quality, Hangzhou 310008, China*

^4^*Zhejiang Tiantong National Station for Forest Ecosystem Research, The Shanghai Key Lab for Urban Ecological Processes and Eco-Restoration, School of Ecological and Environmental Sciences, East China Normal University, Shanghai 200241, China*

^5^*Institute of Eco-Chongming (IEC), East China Normal University, Shanghai 202162, China*

^6^*Department of Microbiology Biocenter 1, Viikinkaari 9 University of Helsinki, Helsinki 00790, Finland*

*^*^Corresponding author*

^*^Corresponding Author: [lx@lzu.edu.cn](mailto:lx@lzu.edu.cn)

**

**

**Figure S1. The process of oomycete quantification, DNA extraction and sequencing.** Quantifying soil oomycetes was performed by inserting a pair of oomycete-specific primers into a vector to constitute a quantitative plasmid and adding at a known concentration into the soil to be extracted. In this process, plasmids and oomycete DNA in the soil were maintained at the same loss rate during the steps of DNA extraction, amplification and sequencing. Absolute abundance of soil oomycetes was finally quantified by calculating the relative ratio of plasmid reads to oomycete reads in the sequencing results.

**
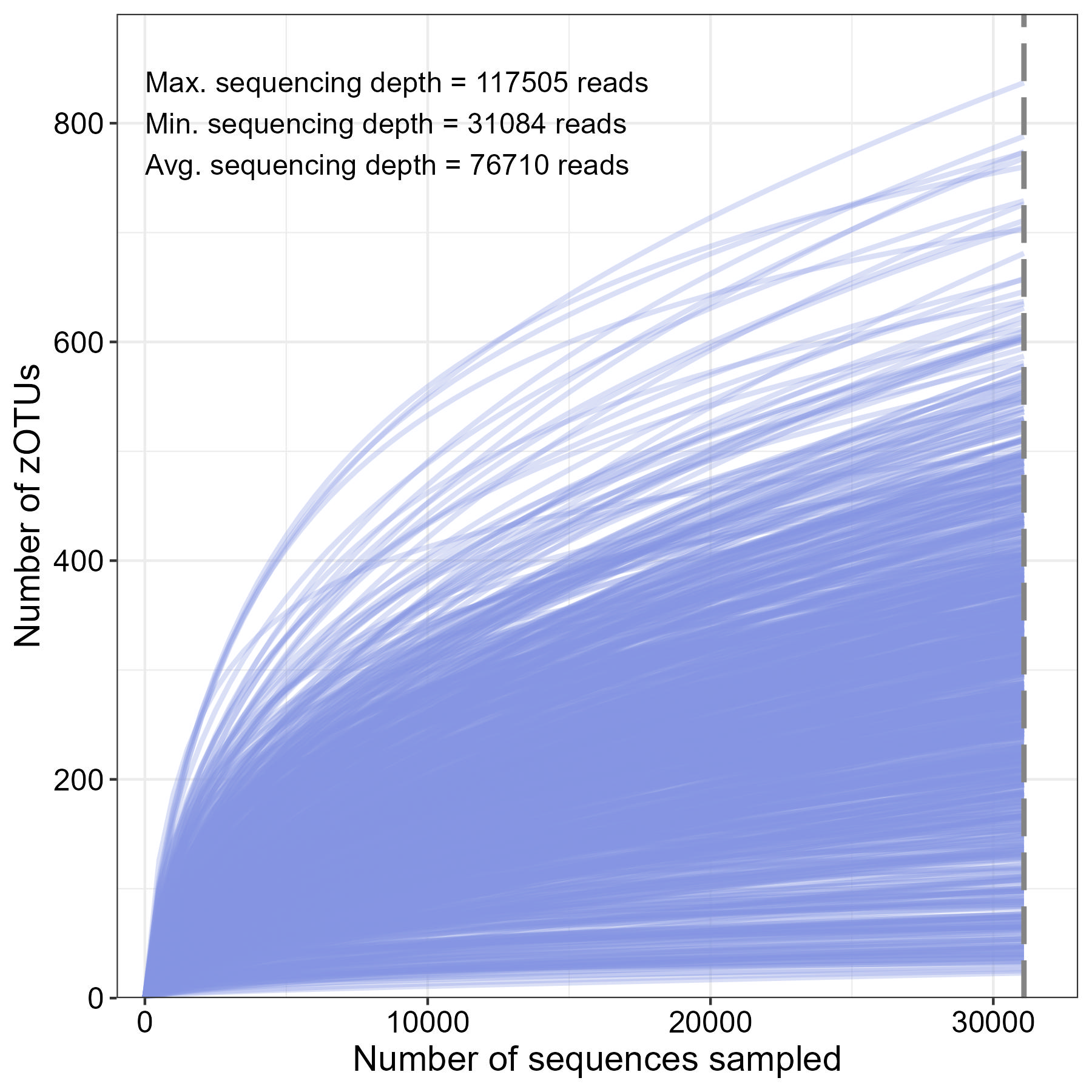
**

**Figure S2. The rarefaction curves of oomycetes in 972 soil samples from 244 study sites across China’s major grassland.** The sequencing depth for all samples ranged from 31,084 to 117,505 reads, with an average of 76,710 reads. The number of reads for all samples was randomized to 31084, and the curves were plotted in steps of 500. The curves become progressively flatter as the number of sequences sampled increases, indicating that the sequencing depth was appropriate.

**
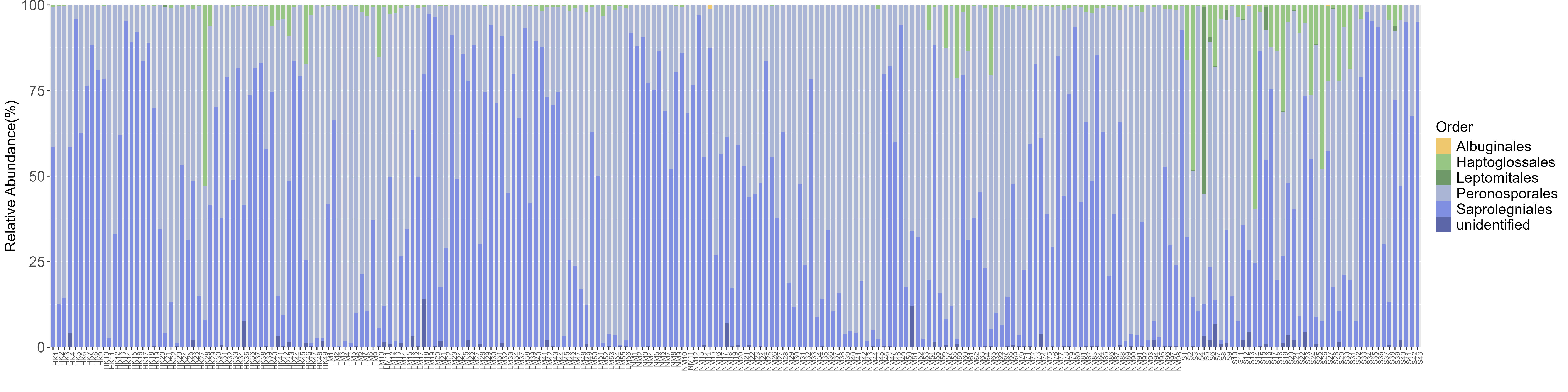
**

**Figure S3. The composition of oomycetes based on the order level in 244 study sites.** The oomycetes detected at the order level consisted of Albuginales, Haptoglossales, Leptomitales, Peronosporales, Saprolegniales, with Peronosporales (60.76%) and Saprolegniales (35.66%) being two of the most abundant orders.


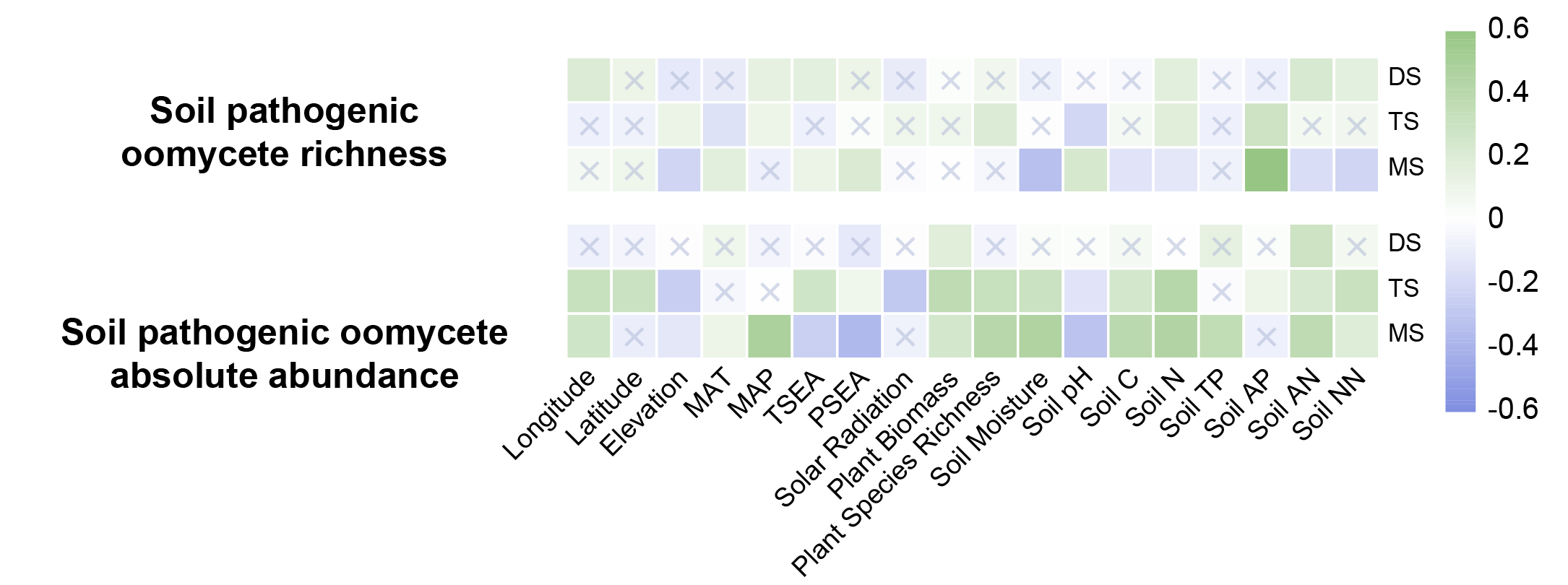


**Figure S4. The responses of soil pathogenic oomycete richness and absolute abundance to 18 biotic and abiotic variables across three different grassland types (DS: desert grassland, TS: Typical grassland, MS: meadow grassland).** The Spearman’s rank correlations between geographic variables, climatic factors, soil properties, plant community characteristics, and response variables for pathogenic oomycetes are presented by green (positive) and purple squares (negative). A cross in the middle of the square indicates that there was no significant correlation (*P* > 0.05) between the two variables. The 18 biotic and abiotic variables include longitude, latitude, elevation, mean annual temperature (MAT), mean annual precipitation (MAP), temperature (TSEA) and precipitation seasonality (PSEA), solar radiation, plant species richness, plant biomass, soil moisture, soil pH, soil carbon (Soil C), soil nitrogen (Soil N), soil total phosphorus (Soil TP), soil available phosphorus (Soil AP), ammonia nitrogen (Soil AN) and nitrate nitrogen (Soil NN).


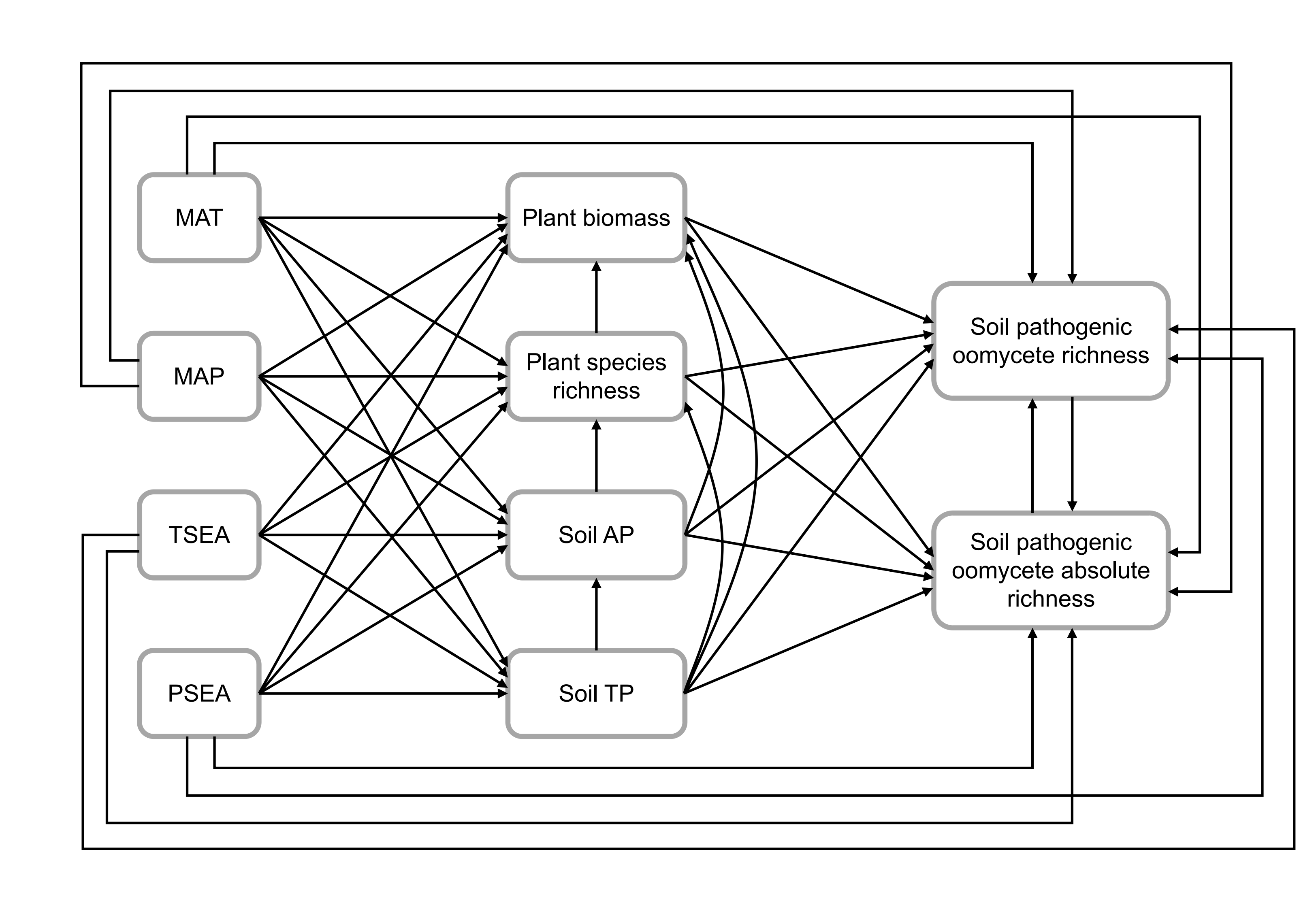


**Figure S5. Prior framework of Bayesian structural equation modeling for richness and absolute abundance of soil pathogenic oomycetes.** The framework hypothesizes the direct and indirect influences of mean annual temperature (MAT), annual precipitation (MAP), temperature seasonality (TSEA), and precipitation seasonality (PSEA) in modulating the richness and absolute abundances of soil pathogenic oomycetes via plant biomass, plant species richness, soil available phosphorus (Soil AP), and soil total phosphorus (Soil TP).

**
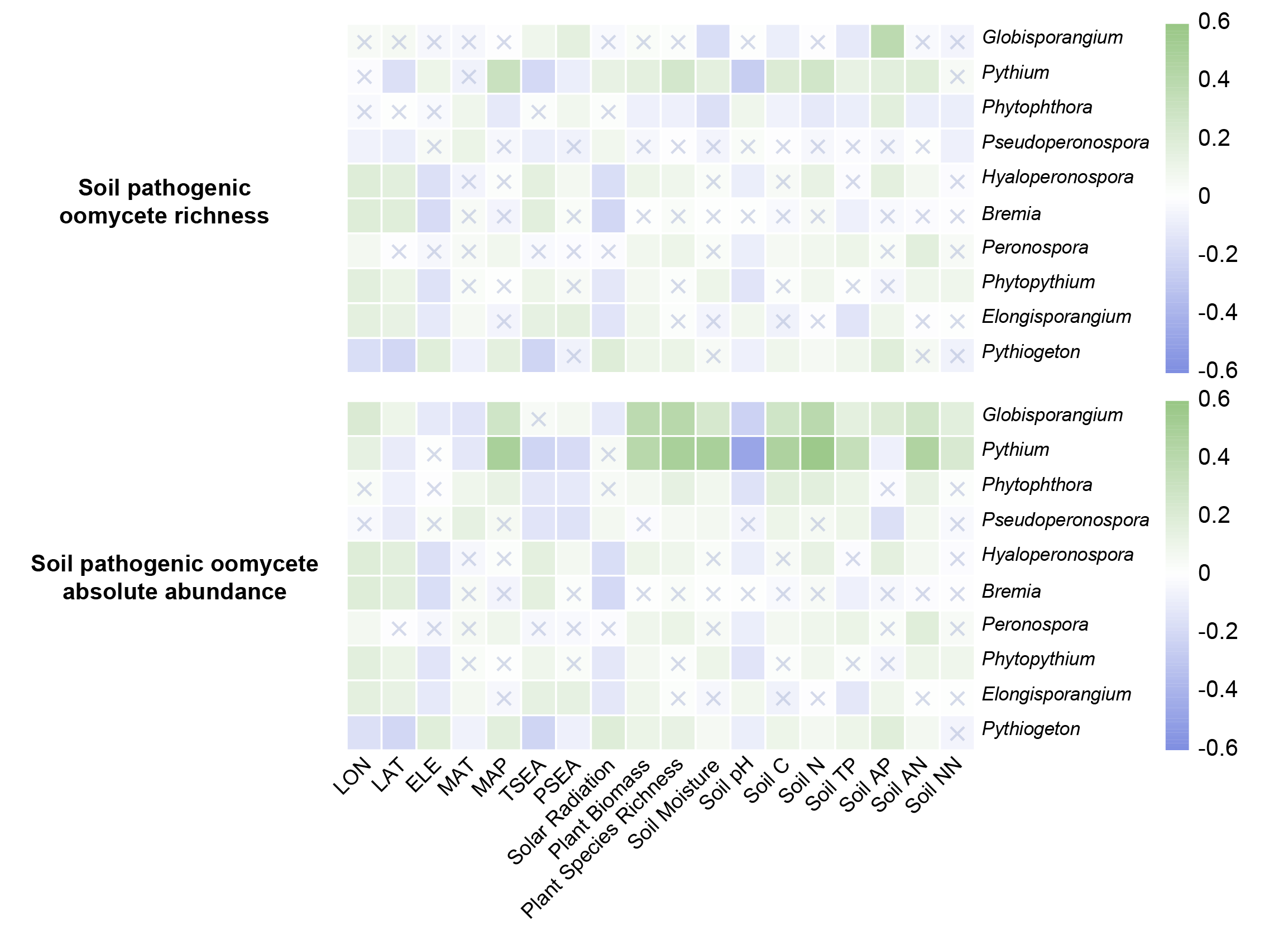
**

**Figure S6. The responses of phytopathogenic oomycete richness and absolute abundance to 18 biotic and abiotic variables by the top 10 soil pathogenic oomycete genera.** The Spearman’s rank correlations between geographic variables, climatic factors, soil properties, plant community characteristics, and response variables for pathogenic oomycetes are presented by green (positive) and purple squares (negative). A cross in the middle of the square indicates that there is no significant correlation (*P* > 0.05) between the pair of variables. The 18 biotic and abiotic variables include longitude, latitude, elevation, mean annual temperature (MAT), mean annual precipitation (MAP), temperature (TSEA) and precipitation seasonality (PSEA), solar radiation, plant species richness, plant biomass, soil moisture, soil pH, soil carbon (Soil C), soil nitrogen (Soil N), soil total phosphorus (Soil TP), soil available phosphorus (Soil AP), ammonia nitrogen (Soil AN) and nitrate nitrogen (Soil NN).


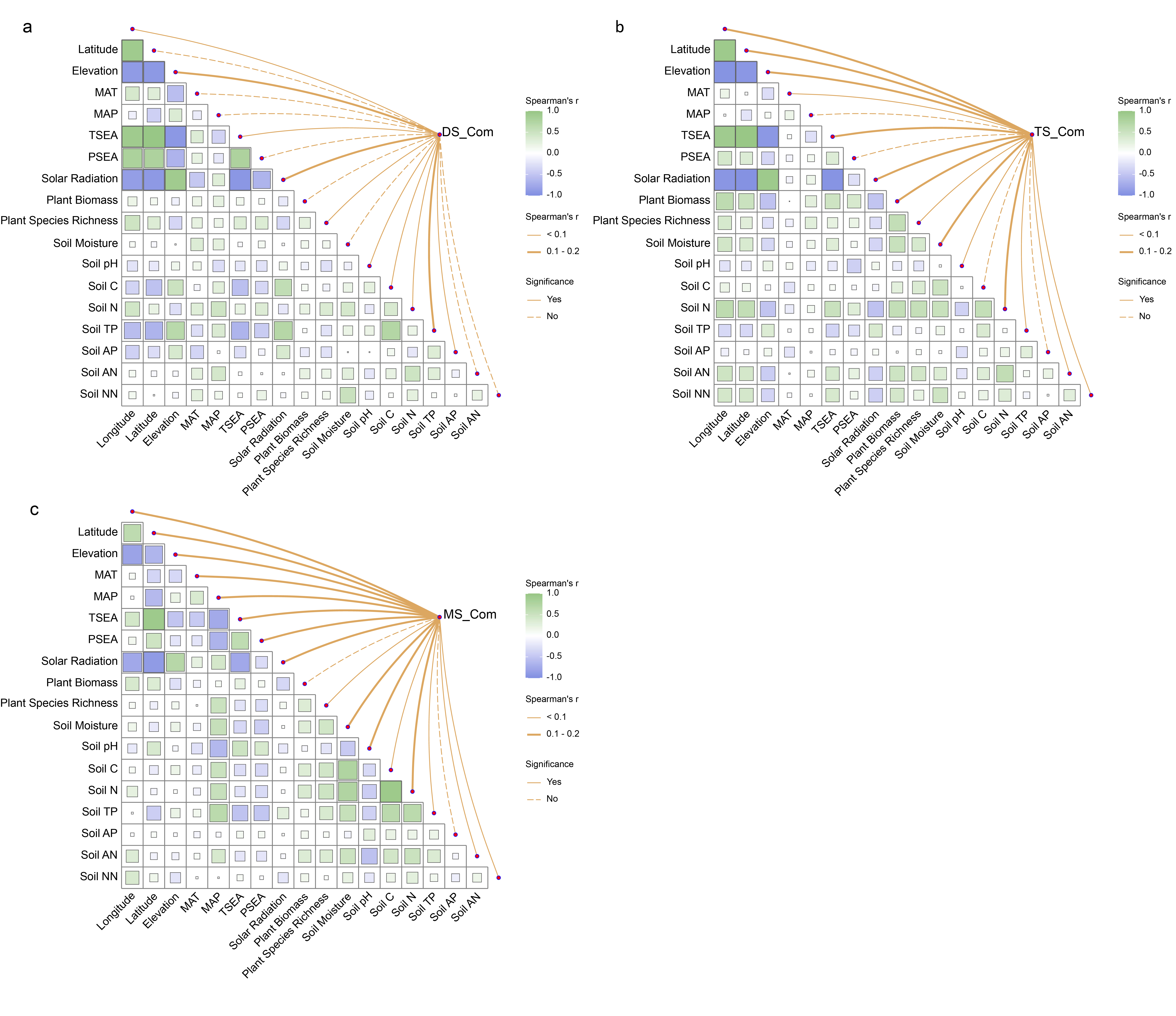


**Figure S7. The Mantel test between 18 biotic and abiotic variables and the matrix of pathogenic oomycete community composition in three different grassland types (DS: desert grassland, TS: typical grassland, MS: meadow grassland).** The size of the boxes in the figure represents the correlation coefficients between environmental variables, with green and purple indicating positive and negative relationships, respectively. Solid and dotted lines indicate significant (*P* < 0.05) and non-significant (*P* > 0.05) correlations, respectively, between soil pathogenic oomycete community composition and the environmental variables, while the width of lines reflect the strength of the Mantel test. The 18 biotic and abiotic variables include longitude, latitude, elevation, mean annual temperature (MAT), mean annual precipitation (MAP), temperature (TSEA) and precipitation seasonality (PSEA), solar radiation, plant species richness, plant biomass, soil moisture, soil pH, soil carbon (Soil C), soil nitrogen (Soil N), soil total phosphorus (Soil TP), soil available phosphorus (Soil AP), ammonia nitrogen (Soil AN) and nitrate nitrogen (Soil NN).


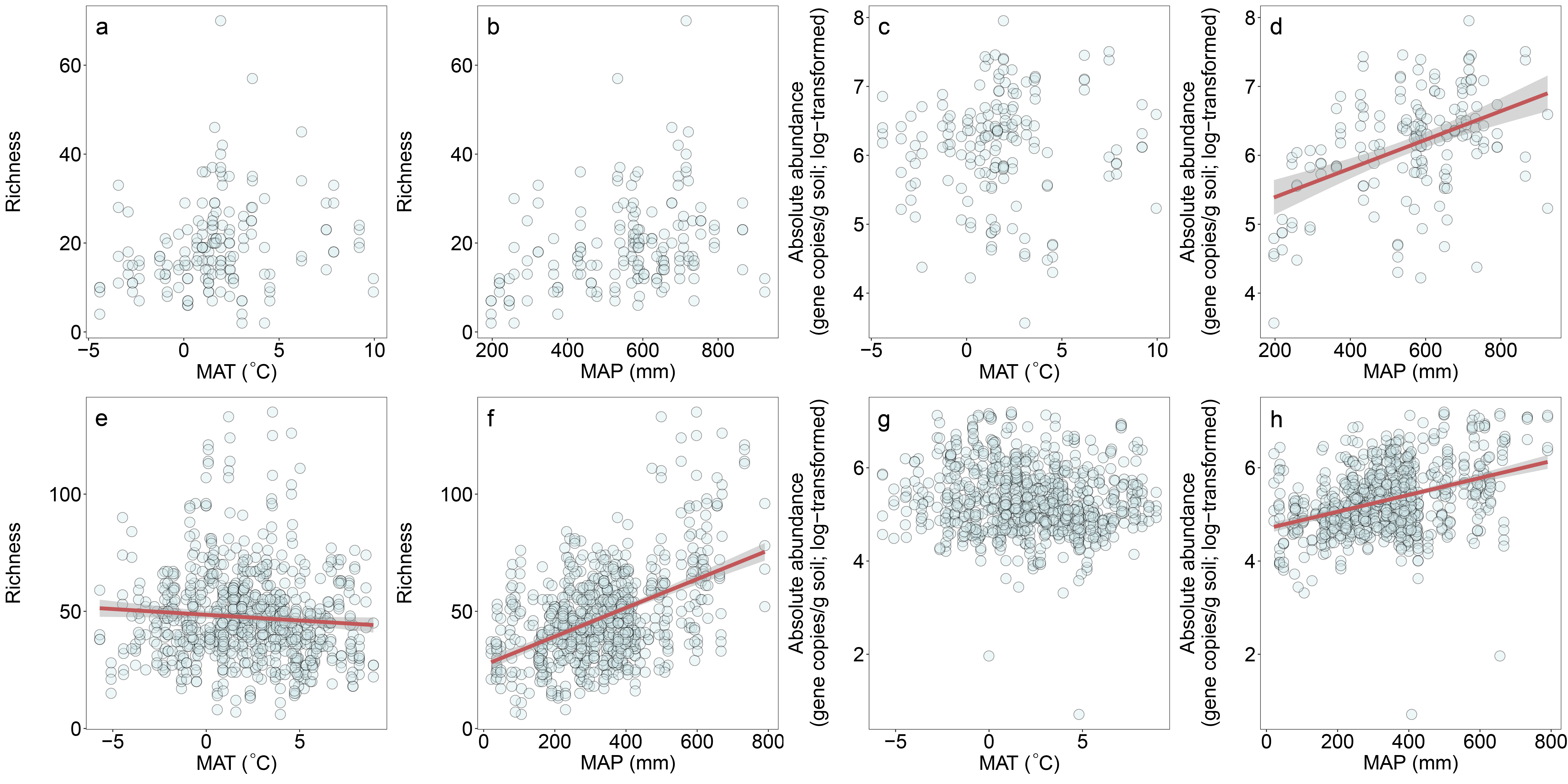


**Figure S8. Effects of mean annual temperature (MAT) and annual precipitation (MAP) for the sampling year on soil pathogenic oomycete richness and absolute abundance.** **a-d** based on climatic data of 2021. **e-h** based on climatic data of 2022. The figures present the results of linear mixed-effects models, including the spatial distance correlation matrix with “Site” as a random effect (n_2021_ = 169, n_2022_ = 803). Regression lines for richness/absolute abundance versus MAT or MAP are fitted to illustrate significant effects (*P* < 0.05), while no fitted lines are shown for non-significant relationships (*P* > 0.05).


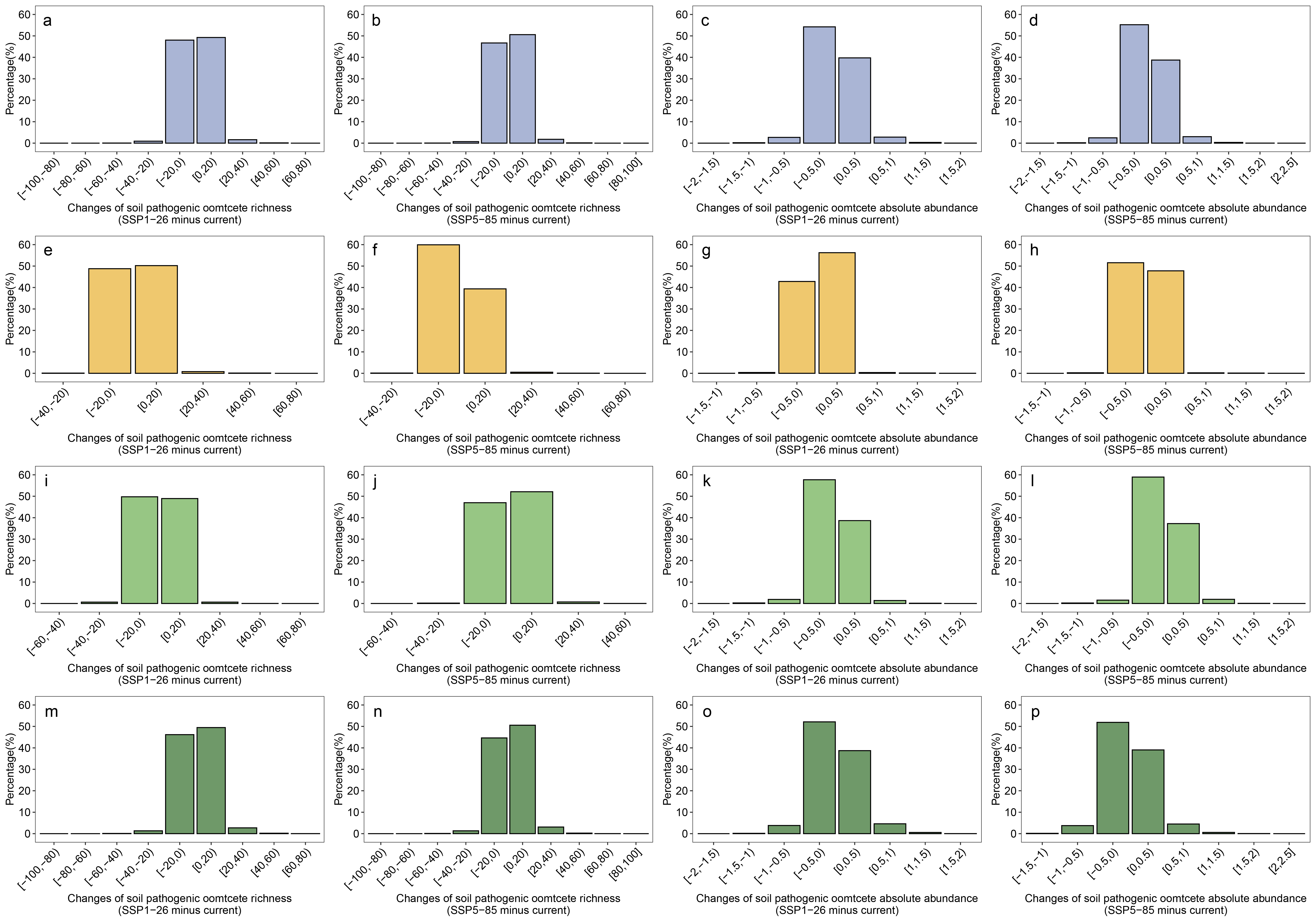


**Figure S9. Regional statistics of differences in richness and absolute abundance of soil pathogenic oomycetes between the SSP1-2.6 and SSP5-8.5 climate scenarios with current conditions. a-d** across main Chinese grasslands; **e-h** across desert grasslands; **i-l** across typical grasslands; **m-p** across meadow grasslands.

**Table S1 Characteristics of 18 biotic and abiotic variables at 972 sample sites across the across China’s main grasslands.** The 18 biotic and abiotic variables include longitude, latitude, elevation, mean annual temperature (MAT), mean annual precipitation (MAP), temperature (TSEA) and precipitation seasonality (PSEA), solar radiation, plant species richness, plant biomass, soil moisture, soil pH, soil carbon (Soil C), soil nitrogen (Soil N), soil total phosphorus (Soil TP), soil available phosphorus (Soil AP), ammonia nitrogen (Soil AN) and nitrate nitrogen (Soil NN).

| **Variables** | **Simplified name** | **Average** | **Standard deviation** | **Range** |
| --- | --- | --- | --- | --- |
| Longitude (°) | Longitude | 102.6274072 | 11.21266431 | 79.02831111 ~ 121.4955778 |
| Latitude (°) | Latitude | 37.1676046 | 5.985927225 | 26.97908333 ~ 50.14539722 |
| Elevation (m) | Elevation | 2754.314609 | 1471.359234 | 403 ~ 4961 |
| Mean Annual Temperature (°C) | MAT | 2.09363855 | 3.057579665 | -5.70000029 ~ 9.94999981 |
| Annual Precipitation (mm) | MAP | 372.8251029 | 174.4851077 | 21 ~ 924 |
| Temperature Seasonality (standard deviation * 100) | TSEA | 1025.867016 | 321.3606055 | 488.9715576 ~ 1719.549805 |
| Precipitation Seasonality (coefficient of variation) | PSEA | 100.5167272 | 18.05990824 | 0 ~ 141.1791382 |
| Net Downward Shortwave Radiation (W m^-2^) | Solar Radiation | 190.1594000 | 25.44047000 | 134.5535 ~ 255.9915 |
| Plant Biomass (g) | Plant Biomass | 33.18976401 | 26.30680739 | 0.5351 ~ 173.80478 |
| Plant Species Richness | Plant Species Richness | 10.00925926 | 7.222156425 | 1 ~ 42 |
| Soil Moisture (%) | Soil Moisture | 0.206385501 | 0.326646192 | 0.00111 ~ 3.680705191 |
| Soil pH | Soil pH | 7.525304527 | 0.858550539 | 5.02 ~ 11.37 |
| Soil Carbon Content (%) | Soil C | 3.359883745 | 3.514522499 | 0.1 ~ 23.437 |
| Soil Nitrogen Content (%) | Soil N | 0.268772634 | 0.269464203 | 0.002 ~ 1.813 |
| Soil Total Phosphorus (‰) | Soil TP | 0.532433208 | 0.347955349 | 0.00188 ~ 2.520955467 |
| Soil Available Phosphorus (mg/kg) | Soil AP | 10.7136853 | 8.223405047 | 0.721891771 ~ 78.19373425 |
| Soil Ammonia Nitrogen (mg/kg) | Soil AN | 8.535973464 | 16.43710009 | 0.000482 ~ 338.4837199 |
| Soil Nitrate Nitrogen (mg/kg) | Soil NN | 5.980163056 | 6.714449122 | 0.0177 ~ 50.22952179 |

**Table S2 Genus name and frequency of oomycetes identified as potential plant pathogens in 972 soil samples across China’s main grasslands.**

| **Genus** | **Frequency** |
| --- | --- |
| *Albugo* | 1 |
| *Bremia* | 20 |
| *Elongisporangium* | 27 |
| *Geolegnia* | 3 |
| *Globisporangium* | 742 |
| *Hyaloperonospora* | 20 |
| *Peronospora* | 22 |
| *Phytophthora* | 311 |
| *Phytopythium* | 12 |
| *Plasmopara* | 5 |
| *Pseudoperonospora* | 44 |
| *Pustula* | 2 |
| *Pythiogeton* | 32 |
| *Pythium* | 289 |
| *Wilsoniana* | 2 |

**Table S3** **The results of Spearman’s rank correlation analysis between** **18 biotic and abiotic variables and** **the richness and absolute abundances of pathogenic oomycetes.** The 18 biotic and abiotic variables include longitude, latitude, elevation, mean annual temperature (MAT), mean annual precipitation (MAP), temperature (TSEA) and precipitation seasonality (PSEA), solar radiation, plant species richness, plant biomass, soil moisture, soil pH, soil carbon (Soil C), soil nitrogen (Soil N), soil total phosphorus (Soil TP), soil available phosphorus (Soil AP), ammonia nitrogen (Soil AN) and nitrate nitrogen (Soil NN), while response variables include the richness and absolute abundance of soil pathogenic oomycetes. The table shows the correlation coefficients between each pair of variables, with significant correlations shown in bold (*P* < 0.05).

| Variables | Richness | Absolute Abundance |
| --- | --- | --- |
| LON | 0.05 | **0.19** |
| LAT | 0.03 | -0.04 |
| ELE | -0.03 | -0.02 |
| MAT | -0.01 | **-0.14** |
| MAP | 0.04 | **0.48** |
| TSEA | 0.06 | **-0.16** |
| PSEA | **0.13** | **-0.16** |
| Solar Radiation | 0.00 | -0.02 |
| Plant Biomass | 0.05 | **0.49** |
| Plant Species Richness | 0.06 | **0.53** |
| Soil Moisture | **-0.14** | **0.51** |
| Soil pH | -0.02 | **-0.41** |
| Soil C | -0.04 | **0.49** |
| Soil N | 0.04 | **0.57** |
| Soil TP | **-0.08** | **0.35** |
| Soil AP | **0.38** | 0.00 |
| Soil AN | 0.01 | **0.45** |
| Soil NN | -0.04 | **0.26** |

**Table S4 The results of Mantel test between 18 biotic and abiotic variables and the matrix of pathogenic oomycete community composition.** The 18 biotic and abiotic variables include longitude, latitude, elevation, mean annual temperature (MAT), mean annual precipitation (MAP), temperature (TSEA) and precipitation seasonality (PSEA), solar radiation, plant species richness, plant biomass, soil moisture, soil pH, soil carbon (Soil C), soil nitrogen (Soil N), soil total phosphorus (Soil TP), soil available phosphorus (Soil AP), ammonia nitrogen (Soil AN) and nitrate nitrogen (Soil NN). The table shows the correlation coefficients and *P*-values of the Mantel test between the variables and the pathogenic oomycete composition matrix, with significant correlations shown in bold (*P* < 0.05).

| Variables | Mantel's *r* | *P* |
| --- | --- | --- |
| Longitude | 0.06 | **0.001** |
| Latitude | 0.15 | **0.001** |
| Elevation | 0.13 | **0.001** |
| MAT | 0.10 | **0.001** |
| MAP | 0.21 | **0.001** |
| TSEA | 0.17 | **0.001** |
| PSEA | 0.01 | 0.358 |
| Solar Radiation | 0.11 | **0.001** |
| Plant Biomass | 0.10 | **0.001** |
| Plant Species Richness | 0.19 | **0.001** |
| Soil Moisture | 0.21 | **0.001** |
| Soil pH | 0.17 | **0.001** |
| Soil C | 0.19 | **0.001** |
| Soil N | 0.22 | **0.001** |
| Soil TP | 0.17 | **0.001** |
| Soil AP | 0.02 | 0.108 |
| Soil AN | 0.13 | **0.001** |
| Soil NN | 0.04 | **0.011** |

**Table S5 Results of parameter estimation by using the** **Bayesian mixed-effects models.** The effects of 13 biotic and abiotic variables on the richness and absolute abundance of soil pathogenic oomycetes. The estimates, estimate errors and 95% confidence intervals for each variable are presented in the table. Significant results are highlighted in bold. The 13 biotic and abiotic variables include mean annual temperature (MAT), mean annual precipitation (MAP), temperature (TSEA) and precipitation seasonality (PSEA), plant species richness, plant biomass, soil moisture, soil pH, soil nitrogen (Soil N), soil total phosphorus (Soil TP), soil available phosphorus (Soil AP), ammonia nitrogen (Soil AN) and nitrate nitrogen (Soil NN).

| **Response** | **Predictor** | **Estimate** | **Est.Error** | **95%CI  (Credible intervals)** |
| --- | --- | --- | --- | --- |
| Soil pathogenic oomycete richness | MAT | -0.05 | 1.34 | -2.71~2.57 |
| Soil pathogenic oomycete richness | MAP | 2.33 | 1.93 | -1.46~6.07 |
| Soil pathogenic oomycete richness | TSEA | 1.32 | 1.84 | -2.27~4.91 |
| Soil pathogenic oomycete richness | PSEA | 1.74 | 1.37 | -0.91~4.46 |
| Soil pathogenic oomycete richness | Plant Biomass | 0.07 | 0.60 | -1.1~1.24 |
| Soil pathogenic oomycete richness | Plant Species Richness | 0.54 | 0.83 | -1.06~2.19 |
| Soil pathogenic oomycete richness | Soil Moisture | -0.40 | 0.90 | -2.19~1.36 |
| Soil pathogenic oomycete richness | Soil pH | 1.68 | 0.94 | -0.17~3.49 |
| Soil pathogenic oomycete richness | Soil N | -0.45 | 1.26 | -2.93~2.02 |
| Soil pathogenic oomycete richness | Soil TP | 1.06 | 0.97 | -0.81~2.98 |
| Soil pathogenic oomycete richness | Soil AP | **2.48** | **0.57** | **1.35~3.58** |
| Soil pathogenic oomycete richness | Soil AN | 0.07 | 0.63 | -1.17~1.3 |
| Soil pathogenic oomycete richness | Soil NN | 0.19 | 0.55 | -0.87~1.26 |
| Soil pathogenic oomycete absolute abundance | MAT | 0.02 | 0.03 | -0.05~0.09 |
| Soil pathogenic oomycete absolute abundance | MAP | **0.17** | **0.06** | **0.06~0.28** |
| Soil pathogenic oomycete absolute abundance | TSEA | **0.13** | **0.05** | **0.03~0.22** |
| Soil pathogenic oomycete absolute abundance | PSEA | -0.04 | 0.03 | -0.1~0.03 |
| Soil pathogenic oomycete absolute abundance | Plant Biomass | **0.05** | **0.03** | **0~0.11** |
| Soil pathogenic oomycete absolute abundance | Plant Species Richness | **0.19** | **0.03** | **0.12~0.26** |
| Soil pathogenic oomycete absolute abundance | Soil Moisture | 0.05 | 0.04 | -0.03~0.12 |
| Soil pathogenic oomycete absolute abundance | Soil pH | -0.04 | 0.04 | -0.11~0.03 |
| Soil pathogenic oomycete absolute abundance | Soil N | 0.02 | 0.05 | -0.08~0.12 |
| Soil pathogenic oomycete absolute abundance | Soil TP | **0.09** | **0.04** | **0.01~0.16** |
| Soil pathogenic oomycete absolute abundance | Soil AP | 0.00 | 0.02 | -0.05~0.04 |
| Soil pathogenic oomycete absolute abundance | Soil AN | 0.04 | 0.03 | -0.01~0.1 |
| Soil pathogenic oomycete absolute abundance | Soil NN | 0.04 | 0.02 | -0.01~0.09 |

**Table S6 Results of contribution estimation by using the Bayesian mixed-effects models.** The effects of 13 biotic and abiotic variables on the richness and absolute abundance of soil pathogenic oomycetes. The estimates, estimated standard deviations and 95% confidence intervals for each variable are presented in the table. The 13 biotic and abiotic variables include mean annual temperature (MAT), mean annual precipitation (MAP), temperature (TSEA) and precipitation seasonality (PSEA), plant species richness, plant biomass, soil moisture, soil pH, soil nitrogen (Soil N), soil total phosphorus (Soil TP), soil available phosphorus (Soil AP), ammonia nitrogen (Soil AN) and nitrate nitrogen (Soil NN).

| **Response** | **Predictor** | **Estimate** | **Est.sd** | **95%CI  (Credible intervals)** |
| --- | --- | --- | --- | --- |
| Soil pathogenic oomycete richness | MAT | 3.17 | 1.29 | 0.64~5.71 |
| Soil pathogenic oomycete richness | MAP | 9.50 | 2.13 | 5.13~13.5 |
| Soil pathogenic oomycete richness | TSEA | 2.78 | 1.62 | 0.16~6.16 |
| Soil pathogenic oomycete richness | PSEA | 2.06 | 1.19 | 0.13~4.54 |
| Soil pathogenic oomycete richness | Plant Biomass | 1.31 | 0.98 | 0.05~3.67 |
| Soil pathogenic oomycete richness | Plant Species Richness | 5.09 | 1.79 | 1.57~8.57 |
| Soil pathogenic oomycete richness | Soil Moisture | 6.73 | 2.11 | 2.48~10.73 |
| Soil pathogenic oomycete richness | Soil pH | 3.01 | 1.54 | 0.25~6.09 |
| Soil pathogenic oomycete richness | Soil N | 2.15 | 1.57 | 0.09~5.77 |
| Soil pathogenic oomycete richness | Soil TP | 5.29 | 1.86 | 1.5~8.86 |
| Soil pathogenic oomycete richness | Soil AP | 18.31 | 1.46 | 15.52~21.22 |
| Soil pathogenic oomycete richness | Soil AN | 1.49 | 1.11 | 0.06~4.13 |
| Soil pathogenic oomycete richness | Soil NN | 1.06 | 0.80 | 0.04~2.96 |
| Soil pathogenic oomycete richness | Error | 38.05 | 2.17 | 34.07~42.57 |
| Soil pathogenic oomycete absolute abundance | MAT | 1.50 | 1.05 | 0.06~3.88 |
| Soil pathogenic oomycete absolute abundance | MAP | 8.80 | 2.37 | 4.13~13.41 |
| Soil pathogenic oomycete absolute abundance | TSEA | 6.91 | 1.70 | 3.44~10.09 |
| Soil pathogenic oomycete absolute abundance | PSEA | 3.12 | 1.33 | 0.52~5.75 |
| Soil pathogenic oomycete absolute abundance | Plant Biomass | 5.18 | 1.68 | 1.87~8.49 |
| Soil pathogenic oomycete absolute abundance | Plant Species Richness | 13.24 | 1.88 | 9.55~16.93 |
| Soil pathogenic oomycete absolute abundance | Soil Moisture | 3.50 | 2.00 | 0.22~7.6 |
| Soil pathogenic oomycete absolute abundance | Soil pH | 2.60 | 1.57 | 0.15~5.98 |
| Soil pathogenic oomycete absolute abundance | Soil N | 2.47 | 1.87 | 0.1~6.89 |
| Soil pathogenic oomycete absolute abundance | Soil TP | 5.94 | 1.91 | 2.04~9.58 |
| Soil pathogenic oomycete absolute abundance | Soil AP | 2.29 | 1.26 | 0.16~4.85 |
| Soil pathogenic oomycete absolute abundance | Soil AN | 2.10 | 1.38 | 0.11~5.13 |
| Soil pathogenic oomycete absolute abundance | Soil NN | 3.39 | 1.36 | 0.71~6.08 |
| Soil pathogenic oomycete absolute abundance | Error | 38.97 | 1.83 | 35.24~42.55 |

**Table S7 Results of the Bayesian structural equation model.** The estimate errors and 95% confidence intervals for each predictor in the Bayesian SEM for pathways to soil pathogenic oomycete richness and absolute abundance. Significant results are highlighted in bold. (MAT = mean annual temperature, MAP = annual precipitation, TSEA = temperature seasonality, PSEA = precipitation seasonality, Soil AP = soil available phosphorus, Soil TP = soil total phosphorus)

| **Response** | **Predictor** | **Estimate** | **Est.Error** | **95%CI  (Credible intervals)** |
| --- | --- | --- | --- | --- |
| Soil pathogenic oomycete richness | MAT | 0.01 | 0.06 | -0.11~0.14 |
| Soil pathogenic oomycete richness | MAP | -0.03 | 0.08 | -0.2~0.13 |
| Soil pathogenic oomycete richness | TSEA | 0.00 | 0.09 | -0.17~0.17 |
| Soil pathogenic oomycete richness | PSEA | 0.09 | 0.07 | -0.03~0.22 |
| Soil pathogenic oomycete richness | Plant Species Richness | -0.03 | 0.04 | -0.1~0.05 |
| Soil pathogenic oomycete richness | Plant Biomass | -0.01 | 0.03 | -0.06~0.05 |
| Soil pathogenic oomycete richness | Soil TP | 0.01 | 0.04 | -0.07~0.09 |
| Soil pathogenic oomycete richness | Soil AP | **0.10** | **0.03** | **0.05~0.15** |
| Soil pathogenic oomycete richness | Soil pathogenic oomycete absolute abundance | **0.23** | **0.02** | **0.18~0.27** |
| Soil pathogenic oomycete absolute abundance | MAT | 0.00 | 0.04 | -0.08~0.08 |
| Soil pathogenic oomycete absolute abundance | MAP | **0.28** | **0.06** | **0.17~0.4** |
| Soil pathogenic oomycete absolute abundance | TSEA | **0.18** | **0.05** | **0.08~0.29** |
| Soil pathogenic oomycete absolute abundance | PSEA | -0.07 | 0.04 | -0.15~0.02 |
| Soil pathogenic oomycete absolute abundance | Plant Species Richness | **0.23** | **0.04** | **0.14~0.31** |
| Soil pathogenic oomycete absolute abundance | Plant Biomass | **0.08** | **0.03** | **0.01~0.14** |
| Soil pathogenic oomycete absolute abundance | Soil TP | **0.17** | **0.04** | **0.09~0.25** |
| Soil pathogenic oomycete absolute abundance | Soil AP | **-0.07** | **0.03** | **-0.13~-0.01** |
| Soil pathogenic oomycete absolute abundance | Soil pathogenic oomycete richness | **0.27** | **0.03** | **0.2~0.34** |
| Plant Biomass | MAT | **-0.16** | **0.05** | **-0.25~-0.07** |
| Plant Biomass | MAP | **0.37** | **0.06** | **0.25~0.49** |
| Plant Biomass | TSEA | **0.33** | **0.06** | **0.21~0.44** |
| Plant Biomass | PSEA | **0.13** | **0.05** | **0.05~0.22** |
| Plant Biomass | Plant Species Richness | **0.23** | **0.04** | **0.15~0.31** |
| Plant Biomass | Soil TP | 0.07 | 0.04 | -0.01~0.15 |
| Plant Biomass | Soil AP | 0.02 | 0.03 | -0.03~0.08 |
| Plant Species Richness | MAT | **-0.26** | **0.04** | **-0.34~-0.17** |
| Plant Species Richness | MAP | **0.64** | **0.06** | **0.53~0.74** |
| Plant Species Richness | TSEA | 0.07 | 0.06 | -0.05~0.19 |
| Plant Species Richness | PSEA | 0.01 | 0.05 | -0.08~0.1 |
| Plant Species Richness | Soil TP | 0.02 | 0.03 | -0.04~0.09 |
| Plant Species Richness | Soil AP | 0.00 | 0.02 | -0.05~0.04 |
| Soil TP | MAT | -0.09 | 0.05 | -0.19~0 |
| Soil TP | MAP | **0.46** | **0.06** | **0.34~0.58** |
| Soil TP | TSEA | **-0.22** | **0.07** | **-0.36~-0.09** |
| Soil TP | PSEA | -0.10 | 0.05 | -0.2~0 |
| Soil AP | MAT | **-0.14** | **0.05** | **-0.24~-0.03** |
| Soil AP | MAP | **-0.13** | **0.07** | **-0.27~0** |
| Soil AP | TSEA | 0.03 | 0.07 | -0.11~0.16 |
| Soil AP | PSEA | -0.01 | 0.05 | -0.12~0.09 |
| Soil AP | Soil TP | **0.33** | **0.05** | **0.24~0.42** |

**Table S8 The results of Spearman’s rank correlation analysis between 18 biotic and abiotic variables and soil pathogenic oomycete richness and absolute abundance in three different grassland types (DS: Desert grassland, TS: Typical grassland, MS: Meadow grassland).** The 18 biotic and abiotic variables include longitude, latitude, elevation, mean annual temperature (MAT), mean annual precipitation (MAP), temperature (TSEA) and precipitation seasonality (PSEA), solar radiation, plant species richness, plant biomass, soil moisture, soil pH, soil carbon (Soil C), soil nitrogen (Soil N), soil total phosphorus (Soil TP), soil available phosphorus (Soil AP), ammonia nitrogen (Soil AN) and nitrate nitrogen (Soil NN). The table shows the correlation coefficients between each pair of variables, with significant correlations shown in bold (*P* < 0.05).

| Variable | Richness | | | Absolute Abundance | | |
| --- | --- | --- | --- | --- | --- | --- |
| Grassland Type | Desert Grassland | Typical Grassland | Meadow Grassland | Desert Grassland | Typical Grassland | Meadow Grassland |
| Longitude | **0.21** | -0.08 | 0.06 | -0.07 | **0.32** | **0.28** |
| Latitude | 0.11 | -0.06 | 0.09 | -0.05 | **0.30** | -0.09 |
| Elevation | -0.11 | **0.12** | **-0.21** | 0.00 | **-0.26** | **-0.12** |
| MAT | -0.10 | **-0.15** | **0.17** | 0.10 | -0.04 | **0.10** |
| MAP | **0.15** | **0.11** | -0.08 | -0.05 | 0.01 | **0.49** |
| TSEA | **0.16** | -0.08 | **0.12** | -0.01 | **0.27** | **-0.25** |
| PSEA | 0.11 | 0.03 | **0.22** | -0.11 | **0.10** | **-0.37** |
| Solar Radiation | -0.09 | 0.10 | -0.01 | 0.00 | **-0.28** | -0.06 |
| Plant Biomass | 0.03 | **0.10** | 0.02 | **0.18** | **0.38** | **0.25** |
| Plant Species Richness | 0.08 | **0.21** | -0.04 | -0.05 | **0.33** | **0.41** |
| Soil Moisture | -0.06 | 0.00 | **-0.33** | 0.04 | **0.30** | **0.47** |
| Soil pH | -0.02 | **-0.21** | **0.24** | 0.02 | **-0.14** | **-0.32** |
| Soil C | -0.03 | 0.06 | **-0.13** | 0.06 | **0.25** | **0.39** |
| Soil N | **0.17** | **0.19** | **-0.12** | 0.01 | **0.43** | **0.45** |
| Soil TP | -0.04 | -0.07 | -0.06 | 0.14 | -0.01 | **0.37** |
| Soil AP | -0.07 | **0.29** | **0.62** | 0.02 | **0.11** | -0.07 |
| Soil AN | **0.23** | 0.07 | **-0.17** | **0.28** | **0.23** | **0.37** |
| Soil NN | **0.15** | 0.09 | **-0.21** | 0.08 | **0.32** | **0.19** |

**Table S9 The results of Mantel test between 18 biotic and abiotic variables and the matrix of pathogenic oomycete community composition in three different grassland types.** The 18 biotic and abiotic variables include longitude, latitude, elevation, mean annual temperature (MAT), mean annual precipitation (MAP), temperature (TSEA) and precipitation seasonality (PSEA), solar radiation, plant species richness, plant biomass, soil moisture, soil pH, soil carbon (Soil C), soil nitrogen (Soil N), soil total phosphorus (Soil TP), soil available phosphorus (Soil AP), ammonia nitrogen (Soil AN) and nitrate nitrogen (Soil NN). The table shows the correlation coefficients between the variables and the soil pathogenic oomycete composition matrix, with significant correlations shown in bold (*P* < 0.05).

| Variable | Desert Grassland | | Typical Grassland | | Meadow Grassland | | | |
| --- | --- | --- | --- | --- | --- | --- | --- | --- |
|  | Mantel's *r* | *P* | Mantel's *r* | *P* | Mantel's *r* | *P* | |  |
| Longitude | 0.080 | **0.015** | 0.150 | **0.001** | 0.163 | | **0.001** | |
| Latitude | 0.050 | 0.061 | 0.184 | **0.001** | 0.169 | | **0.001** | |
| Elevation | 0.103 | **0.004** | 0.165 | **0.001** | 0.173 | | **0.001** | |
| MAT | 0.049 | 0.103 | 0.097 | **0.001** | 0.126 | | **0.001** | |
| MAP | 0.064 | 0.076 | 0.034 | 0.097 | 0.199 | | **0.001** | |
| TSEA | 0.057 | **0.009** | 0.190 | **0.001** | 0.174 | | **0.001** | |
| PSEA | 0.039 | 0.129 | -0.025 | 0.803 | 0.124 | | **0.001** | |
| Solar Radiation | 0.127 | **0.001** | 0.136 | **0.001** | 0.150 | | **0.001** | |
| Plant Biomass | 0.037 | 0.213 | 0.122 | **0.001** | 0.012 | | 0.220 | |
| Plant Species Richness | 0.070 | **0.027** | 0.099 | **0.001** | 0.056 | | **0.001** | |
| Soil Moisture | -0.025 | 0.710 | 0.102 | **0.001** | 0.152 | | **0.001** | |
| Soil pH | 0.061 | **0.036** | 0.038 | **0.041** | 0.185 | | **0.001** | |
| Soil C | 0.083 | **0.034** | 0.007 | 0.416 | 0.087 | | **0.001** | |
| Soil N | 0.075 | **0.047** | 0.108 | **0.001** | 0.108 | | **0.001** | |
| Soil TP | 0.122 | **0.001** | 0.065 | **0.005** | 0.097 | | **0.001** | |
| Soil AP | 0.079 | **0.046** | 0.001 | 0.502 | -0.004 | | 0.603 | |
| Soil AN | -0.028 | 0.720 | 0.078 | **0.002** | 0.072 | | **0.001** | |
| Soil NN | -0.089 | 0.974 | 0.077 | **0.001** | 0.066 | | **0.001** | |

**Table S10 The results of Spearman’s rank correlation analysis between 18 biotic and abiotic variables and richness for the dominant genera of soil pathogenic oomycetes.** The 18 biotic and abiotic variables include longitude, latitude, elevation, mean annual temperature (MAT), mean annual precipitation (MAP), temperature (TSEA) and precipitation seasonality (PSEA), solar radiation, plant species richness, plant biomass, soil moisture, soil pH, soil carbon (Soil C), soil nitrogen (Soil N), soil total phosphorus (Soil TP), soil available phosphorus (Soil AP), ammonia nitrogen (Soil AN) and nitrate nitrogen (Soil NN). The table shows the correlation coefficients between each pair of variables, with significant ones in bold (*P* < 0.05).

| Variables | *Globisporangium* | *Pythium* | *Phytophthora* | *Pseudoperonospora* | *Hyaloperonospora* | *Bremia* | *Peronospora* | *Phytopythium* | *Elongisporangium* | *Pythiogeton* |
| --- | --- | --- | --- | --- | --- | --- | --- | --- | --- | --- |
| LON | 0.04 | -0.01 | -0.03 | **-0.07** | **0.19** | **0.20** | **0.07** | **0.17** | **0.16** | **-0.17** |
| LAT | 0.06 | **-0.16** | 0.01 | **-0.08** | **0.16** | **0.18** | 0.00 | **0.12** | **0.14** | **-0.21** |
| ELE | -0.03 | **0.11** | -0.03 | 0.05 | **-0.16** | **-0.18** | -0.04 | **-0.14** | **-0.11** | **0.19** |
| MAT | -0.04 | -0.06 | **0.09** | **0.12** | -0.04 | 0.04 | 0.05 | 0.03 | **0.07** | **-0.07** |
| MAP | 0.00 | **0.31** | **-0.11** | -0.03 | 0.03 | -0.05 | **0.09** | 0.01 | -0.04 | **0.16** |
| TSEA | **0.09** | **-0.19** | 0.03 | **-0.08** | **0.16** | **0.17** | -0.03 | **0.10** | **0.15** | **-0.22** |
| PSEA | **0.16** | **-0.09** | **0.08** | -0.06 | **0.07** | 0.03 | -0.01 | 0.05 | **0.15** | -0.06 |
| Solar Radiation | -0.02 | **0.14** | 0.02 | **0.08** | **-0.17** | **-0.20** | -0.01 | **-0.12** | **-0.13** | **0.20** |
| Plant Biomass | 0.03 | **0.16** | **-0.08** | -0.04 | **0.11** | 0.01 | **0.09** | **0.07** | **0.10** | **0.11** |
| Plant Species Richness | 0.02 | **0.25** | **-0.08** | 0.00 | **0.10** | 0.04 | **0.11** | 0.03 | 0.03 | **0.12** |
| Soil Moisture | **-0.17** | **0.16** | **-0.16** | -0.05 | 0.04 | 0.01 | 0.04 | **0.11** | -0.05 | 0.05 |
| Soil pH | 0.01 | **-0.25** | **0.10** | 0.03 | **-0.09** | 0.01 | **-0.08** | **-0.13** | **0.08** | **-0.07** |
| Soil C | **-0.08** | **0.21** | **-0.07** | 0.00 | 0.05 | -0.03 | **0.06** | 0.02 | -0.06 | **0.10** |
| Soil N | 0.00 | **0.27** | **-0.10** | -0.04 | **0.13** | 0.05 | **0.09** | **0.09** | 0.00 | **0.07** |
| Soil TP | **-0.11** | **0.14** | **-0.09** | -0.01 | -0.01 | **-0.08** | **0.11** | 0.02 | **-0.13** | **0.10** |
| Soil AP | **0.39** | **0.17** | **0.17** | -0.03 | **0.15** | -0.03 | 0.04 | -0.03 | **0.09** | **0.18** |
| Soil AN | -0.03 | **0.18** | **-0.09** | 0.01 | **0.07** | -0.01 | **0.17** | **0.10** | 0.00 | 0.06 |
| Soil NN | -0.05 | 0.05 | **-0.08** | **-0.08** | -0.01 | 0.00 | 0.05 | **0.09** | 0.02 | -0.06 |

**Table S11 The results of Spearman’s rank correlation analysis between 18 biotic and abiotic variables and absolute abundance for the dominant genera of soil pathogenic oomycetes.** The 18 biotic and abiotic variables include longitude, latitude, elevation, mean annual temperature (MAT), mean annual precipitation (MAP), temperature (TSEA) and precipitation seasonality (PSEA), solar radiation, plant species richness, plant biomass, soil moisture, soil pH, soil carbon (Soil C), soil nitrogen (Soil N), soil total phosphorus (Soil TP), soil available phosphorus (Soil AP), ammonia nitrogen (Soil AN) and nitrate nitrogen (Soil NN). The table shows the correlation coefficients between each pair of variables, with significant ones in bold (*P* < 0.05).

| Variables | *Globisporangium* | *Pythium* | *Phytophthora* | *Pseudoperonospora* | *Hyaloperonospora* | *Bremia* | *Peronospora* | *Phytopythium* | *Elongisporangium* | *Pythiogeton* |
| --- | --- | --- | --- | --- | --- | --- | --- | --- | --- | --- |
| LON | **0.22** | **0.14** | 0.04 | -0.03 | **0.20** | **0.19** | **0.08** | **0.17** | **0.15** | **-0.16** |
| LAT | **0.11** | **-0.10** | **-0.07** | **-0.09** | **0.17** | **0.17** | 0.00 | **0.12** | **0.13** | **-0.21** |
| ELE | **-0.11** | 0.01 | 0.00 | 0.04 | **-0.16** | **-0.17** | -0.04 | **-0.14** | **-0.11** | **0.19** |
| MAT | **-0.14** | **-0.12** | **0.10** | **0.15** | -0.04 | 0.05 | 0.06 | 0.03 | **0.07** | **-0.07** |
| MAP | **0.28** | **0.50** | **0.13** | 0.06 | 0.03 | -0.04 | **0.10** | 0.02 | -0.04 | **0.17** |
| TSEA | 0.05 | **-0.22** | **-0.12** | **-0.13** | **0.16** | **0.16** | -0.04 | **0.10** | **0.15** | **-0.22** |
| PSEA | **0.07** | **-0.18** | **-0.10** | **-0.15** | **0.07** | 0.02 | -0.03 | 0.04 | **0.15** | **-0.07** |
| Solar Radiation | **-0.11** | 0.04 | 0.05 | **0.08** | **-0.17** | **-0.20** | -0.01 | **-0.12** | **-0.13** | **0.20** |
| Plant Biomass | **0.39** | **0.42** | **0.08** | -0.01 | **0.11** | 0.01 | **0.10** | **0.07** | **0.10** | **0.12** |
| Plant Species Richness | **0.41** | **0.50** | **0.14** | **0.07** | **0.10** | 0.04 | **0.12** | 0.03 | 0.02 | **0.13** |
| Soil Moisture | **0.24** | **0.50** | **0.08** | **0.08** | 0.04 | 0.01 | 0.05 | **0.11** | -0.04 | **0.06** |
| Soil pH | **-0.23** | **-0.47** | **-0.14** | -0.04 | **-0.09** | 0.01 | **-0.09** | **-0.13** | **0.08** | **-0.08** |
| Soil C | **0.28** | **0.48** | **0.17** | **0.10** | 0.05 | -0.02 | **0.08** | 0.02 | -0.05 | **0.10** |
| Soil N | **0.40** | **0.57** | **0.17** | 0.06 | **0.13** | 0.05 | **0.10** | **0.09** | 0.00 | **0.08** |
| Soil TP | **0.15** | **0.34** | **0.12** | **0.11** | -0.01 | **-0.07** | **0.12** | 0.02 | **-0.12** | **0.10** |
| Soil AP | **0.21** | **-0.08** | -0.01 | **-0.16** | **0.15** | -0.03 | 0.03 | -0.04 | **0.09** | **0.18** |
| Soil AN | **0.26** | **0.46** | **0.13** | **0.08** | **0.07** | -0.01 | **0.18** | **0.10** | 0.01 | **0.07** |
| Soil NN | **0.16** | **0.23** | 0.02 | -0.02 | -0.01 | 0.00 | 0.05 | **0.09** | 0.01 | -0.05 |

**Table S12 The results of** **linear mixed-effects models for effects of mean annual temperature (MAT) and annual precipitation (MAP) for the sampling year on soil pathogenic oomycete richness and absolute abundance (n_2021_ = 169, n_2022_ = 803).** The estimates, standard error, *Z*-value and *P*-value for each climate variable are presented in the table. Significant results are highlighted in bold.

| Year | Response | Climate variable | Estimate | Std. Error | *Z*-value | *P* |
| --- | --- | --- | --- | --- | --- | --- |
| 2021 | Richness | MAT | 1.677 | 1.241 | 1.350 | 0.180 |
|  | Richness | MAP | 1.534 | 1.230 | 1.250 | 0.210 |
|  | Absolute Abundance | MAT | 0.006 | 0.099 | 0.060 | 0.950 |
|  | Absolute Abundance | MAP | 0.254 | 0.089 | 2.850 | **0.004** |
| 2022 | Richness | MAT | -3.286 | 1.247 | -2.640 | **0.008** |
|  | Richness | MAP | 8.891 | 1.140 | 7.800 | **<0.001** |
|  | Absolute Abundance | MAT | -0.061 | 0.043 | -1.430 | 0.150 |
|  | Absolute Abundance | MAP | 0.261 | 0.039 | 6.640 | **<0.001** |

**Table S13 The results of linear mixed-effects models for effects of mean annual temperature (MAT), mean annual precipitation (MAP), temperature (TSEA) and precipitation seasonality (PSEA), and their interactions on soil pathogenic oomycete richness and absolute abundance.** The estimates, standard error, *Z*-value and *P*-value for each climate variable are presented in the table. Significant results are highlighted in bold.

| Variables | Richness | | | | Absolute Abundance | | | |
| --- | --- | --- | --- | --- | --- | --- | --- | --- |
|  | Estimate | Std. Error | *Z*-value | *P* | Estimate | Std. Error | *Z*-value | *P* |
| MAT | -0.66 | 1.40 | -0.47 | 0.640 | -0.055 | 0.036 | -1.520 | 0.130 |
| MAP | 0.54 | 1.48 | 0.36 | 0.720 | 0.388 | 0.038 | 10.270 | **<0.001** |
| MAT:MAP | 1.73 | 1.34 | 1.29 | 0.200 | 0.005 | 0.035 | 0.130 | 0.900 |
| MAT | -0.76 | 1.42 | -0.54 | 0.590 | -0.101 | 0.043 | -2.360 | **0.018** |
| TSEA | 0.29 | 1.56 | 0.19 | 0.850 | -0.120 | 0.046 | -2.600 | **0.009** |
| MAT:TSEA | -1.74 | 1.60 | -1.09 | 0.280 | -0.197 | 0.048 | -4.120 | **<0.001** |
| MAT | -0.22 | 1.39 | -0.16 | 0.870 | -0.075 | 0.043 | -1.720 | 0.085 |
| PSEA | 1.66 | 1.45 | 1.14 | 0.250 | -0.103 | 0.045 | -2.260 | **0.024** |
| MAT:PSEA | 0.58 | 1.42 | 0.41 | 0.680 | -0.075 | 0.044 | -1.690 | 0.091 |
| MAP | 4.04 | 2.21 | 1.83 | 0.068 | 0.428 | 0.055 | 7.770 | **<0.001** |
| TSEA | 2.03 | 1.84 | 1.10 | 0.270 | 0.133 | 0.046 | 2.920 | **0.004** |
| MAP:TSEA | 3.69 | 2.22 | 1.66 | 0.097 | -0.048 | 0.055 | -0.870 | 0.380 |
| MAP | 3.35 | 1.69 | 1.98 | **0.048** | 0.376 | 0.044 | 8.590 | **<0.001** |
| PSEA | 2.49 | 1.41 | 1.77 | 0.077 | -0.010 | 0.037 | -0.260 | 0.790 |
| MAP:PSEA | 3.90 | 1.72 | 2.26 | **0.024** | -0.028 | 0.046 | -0.610 | 0.550 |
| TSEA | -0.13 | 1.65 | -0.08 | 0.940 | -0.085 | 0.050 | -1.700 | 0.089 |
| PSEA | 1.39 | 1.46 | 0.95 | 0.340 | -0.065 | 0.045 | -1.420 | 0.150 |
| TSEA:PSEA | 0.48 | 1.09 | 0.44 | 0.660 | 0.075 | 0.034 | 2.200 | **0.028** |
